# Effects of electron transfer on the stability of hydrogen bonds†
†Electronic supplementary information (ESI) available. See DOI: 10.1039/c7sc03361c



**DOI:** 10.1039/c7sc03361c

**Published:** 2017-08-30

**Authors:** Tyler M. Porter, Gavin P. Heim, Clifford P. Kubiak

**Affiliations:** a Department of Chemistry and Biochemistry , University of California San Diego , 9500 Gilman Drive , La Jolla , California 92093-0358 , USA . Email: ckubiak@ucsd.edu

## Abstract

The measurement of the dimerization constants of hydrogen-bonded ruthenium complexes (**1**_2_, **2**_2_, **3**_2_) linked by a self-complementary pair of 4-pyridylcarboxylic acid ligands in different redox states is reported. Using a combination of FTIR and UV/vis/NIR spectroscopies, the dimerization constants (*K*_D_) of the isovalent, neutral states, **1**_2_, **2**_2_, **3**_2_, were found to range from 75 to 130 M^–1^ (Δ*G* ^0^ = –2.56 to –2.88 kcal mol^–1^), while the dimerization constants (*K*_2–_) of the isovalent, doubly-reduced states, (**1**_2_)^2–^, (**2**_2_)^2–^, (**3**_2_)^2–^, were found to range from 2000 to 2500 M^–1^ (Δ*G* ^0^ = –4.5 to –4.63 kcal mol^–1^). From the aforementioned values and the comproportionation constant for the mixed-valent dimers, the dimerization constants (*K*_MV_) of the mixed-valent, hydrogen-bonded dimers, (**1**_2_)^–^, (**2**_2_)^–^, (**3**_2_)^–^, were found to range from 0.5 × 10^6^ to 1.2 × 10^6^ M^–1^ (Δ*G* ^0^ = –7.78 to –8.31 kcal mol^–1^). On average, the hydrogen-bonded, mixed-valent states are stabilized by –5.27 (0.04) kcal mol^–1^ relative to the isovalent, neutral, hydrogen-bonded dimers and –3.47 (0.06) kcal mol^–1^ relative to the isovalent, dianionic hydrogen bonded dimers. Electron exchange in the mixed valence states imparts significant stability to hydrogen bonding. This is the first quantitative measurement of the strength of hydrogen bonds in the presence and absence of electronic exchange.

## Abstract 2

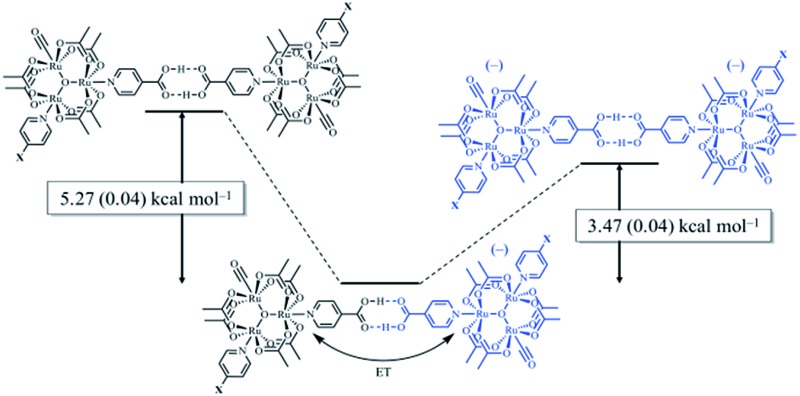
The measurement of the dimerization constants of hydrogen-bonded ruthenium complexes (**1**_2_, **2**_2_, **3**_2_) linked by a self-complementary pair of 4-pyridylcarboxylic acid ligands in different redox states is reported.

## Main text

Electron transfer reactions are among the simplest yet most important reactions in chemistry and biology. The transfer of electrons lies at the heart of any chemical reaction and all biological energy transformations fundamentally depend on electron transfer through proteins and protein assemblies. In the last several decades, extensive experimental and theoretical investigations have been performed to elucidate the nature of electron transfer (ET) in biological energy transfer processes.^1–14^ Electron flow through proteins typically occurs in a site-to-site manner between redox centers separated by distances of 10 to 20 Å.^12,13^ Larger distances require coupling several of these site-to-site reactions such that distances upwards of 25 Å can be traversed.^2,6,12–14^ ET multistep mechanisms are often mediated by intervening amino acid side chains where donor–acceptor ET is favored over tunneling across bridges.^2,13^ ET across such groups typically proceeds across weak, non-covalent interactions as demonstrated by Gray *et al.* in work on mutant azurins.^7,8,12,13^

The study of ET processes across weak, non-covalent interactions thus has important implications in understanding the nature of long range ET in biological systems, but the importance of non-covalent interactions also extends throughout the chemical sciences and affects the stability of artificial supramolecular structures,^15,16^ and selectivity of catalysts.^17–24^ In this report, we examine the fundamental relationship between non-covalent molecular interactions and ET to gain new understanding of electron transfer processes ubiquitous in biological and artificial systems.^2,6–9,12,24–33^

**Fig. 1:**
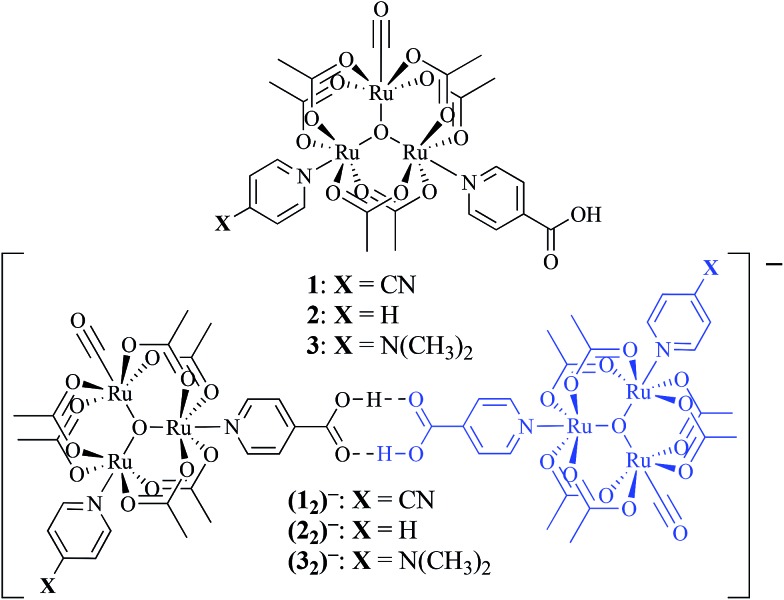
(top) Oxo-centered triruthenium cluster of the type [Ru_3_(μ_3_-O)(OAc)_6_(CO)(L_1_)(ina)] where L_1_ = 4-cyanopyridine (cpy, **1**), pyridine (py, **2**), or 4-dimethylaminopyridine (dmap, **3**) and ina = isonicotinic acid. (bottom) Dimerization interaction upon a one electron reduction to generate the hydrogen-bonded, mixed-valent ions, (**1**_2_)^–^, (**2**_2_)^–^, (**3**_2_)^–^.

While several examples of hydrogen-bonded mixed valency have emerged over the last decade,^27,34–37^ our laboratory has focused on oxo-centered triruthenium clusters featuring isonicotinic acid as a bridging ligand (Fig. 1). Near-IR (NIR) spectroscopic analysis showed the appearance of intervalence charge transfer (IVCT) bands in the singly reduced, hydrogen-bonded dimers, (**1**_2_)^–^, (**2**_2_)^–^, (**3**_2_)^–^, indicative of moderately coupled mixed-valent anions.^38,39^ In an effort to better understand the nature of ET across weak, non-covalent interactions, we compared the strength of hydrogen bonds in dimers of **1–3** in the presence and absence of electron exchange.

**Fig. 2:**
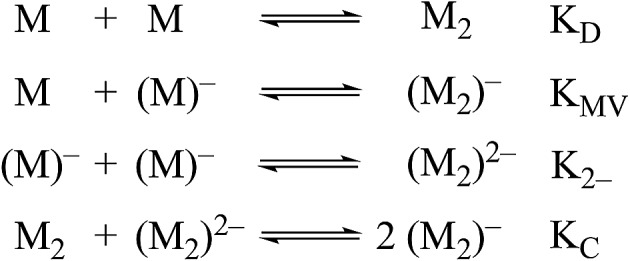
Dimerization equilibria of non-covalent, mixed-valent complexes.

Non-covalent, mixed-valent complexes such as (**1**_2_)^–^, (**2**_2_)^–^, (**3**_2_)^–^, can be described in general by four dimerization equilibria (Fig. 2). Here *K*_D_ and *K*_2–_ are the two isovalent equilibrium constants, which describe the self-dimerization of the neutral and one-electron reduced clusters respectively, *K*_C_ is the comproportionation constant, and *K*_MV_ is the equilibrium dimerization constant of the mixed-valent state. These terms offer thermodynamic information on the formation and stability of hydrogen-bonded species in the three possible redox states. The direct comparison of *K*_MV_ to *K*_D_ or *K*_2–_ allows determination of the relative degree of stability gained from charge transfer across a hydrogen bond.

While several spectroscopic methods for the determination of association constants have been established,^24,40–44^ it is clear that determination of any three of the constants, *K*_MV_, *K*_C_, *K*_2–_ and *K*_D,_ provides the fourth by eqn (1).1
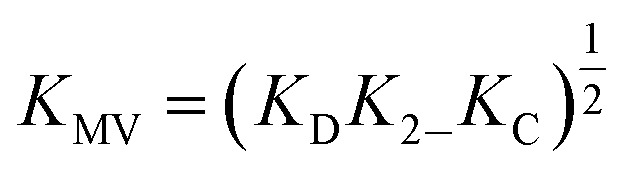



*K*_D_, *K*_2–_, and *K*_C_ can be readily obtained from established spectroscopic and electrochemical methods. The neutral dimerization constant (*K*_D_) was measured by FTIR spectroscopy as the acidic proton of complexes **1–3** was not resolvable in the ^1^H NMR but the *ν*(COOH) bands for the monomer (1748 cm^–1^) and dimer species (1711 cm^–1^) were well resolved in the FTIR spectrum in methylene chloride (DCM) at 25 °C (Fig. S1–S3†).^24,40–44^ Using a variable path length, CaF_2_ windowed cell set to 2.0 mm, the FTIR spectra of complexes **1–3**, and their hydrogen-bonded dimers were recorded in DCM across a range of concentrations from 2.3 mM to 0.25 mM. After solvent subtraction, the *ν*(COOH) bands of the monomeric (1748 cm^–1^) and dimeric (1711 cm^–1^) complexes were fit as two, well resolved Gaussian functions (Fig. S4–S6†) to obtain the integrated spectral area of each band (Table S1†). *K*_D_ was then determined from the eqn (2) where a 1 : 1 self-dimerization model was used (Fig. S7†).^40^
2
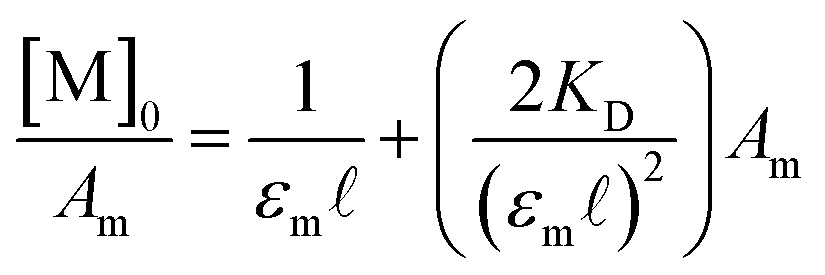



Here, [M]_0_ is the stoichiometric concentration of the solute, *A*_m_ is the integrated spectral area of the monomer band, *ε*_m_ is the extinction coefficient and 𝓁 the cell path length.^40^ Previous studies have shown that the electronic couplings in complexes (**1**_2_)^–^, (**2**_2_)^–^, (**3**_2_)^–^, and Ru_3_O clusters in general have a large dependence on the electron-donating nature of the ancillary pyridine ligand.^45–48^ While similar trends in the equilibrium dimerization constant would be expected, no general trend in *K*_D_ is observed and the dimerization constants remain largely independent of the ancillary ligand (Table 1, *K*_D_ (M^–1^): **1**: 119 (6), **2**: 75 (5), **3**: 130 (8)). In addition to treatment of the monomer band, *K*_D_ can also be determined by consideration of the dimer band through eqn (3).3
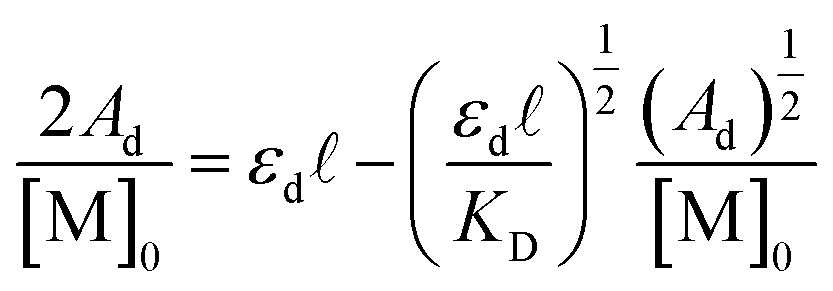



**Table 1:** Equilibrium dimerization constants for complexes **1–3** in DCM at 25 °C

Complex	*K*_D_ (M^–1^)	*K*_2–_ ^*a*^ (10^3^ M^–1^)	*K*_C_ (10^6^)	*K*_MV_ (10^6^ M^–1^)	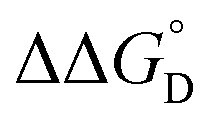 ^*b*^ (kcal mol^–1^)	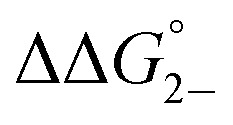 ^*b*^ (kcal mol^–1^)
**1**	119 (6)	2.0 (0.4)	1.09 (0.04)	0.5 (0.1)	–4.95 (0.07)	–3.3 (0.1)
**2**	75 (5)	2.2 (0.3)	3.2 (0.1)	0.7 (0.1)	–5.4 (0.1)	–3.4 (0.1)
**3**	130 (8)	2.5 (0.3)	4.8 (0.2)	1.2 (0.1)	–5.43 (0.06)	–3.68 (0.08)

^*a*^Value for *K*_2–_ was only determined in THF solutions with Co(cp*)_2_ used as a chemical reductant.

^*b*^ 
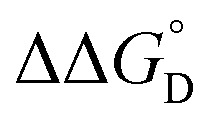
 = 
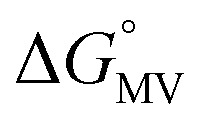
 – 
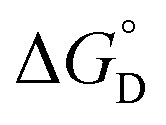
 and 
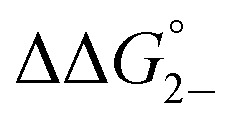
 = 
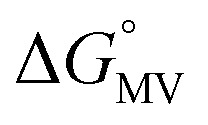
 – 
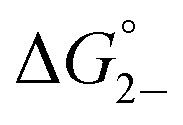
.

While calculation of *K*_D_ should remain independent of band choice, when the dimer *ν*(COOH) band is used (Fig. S7†), a larger degree of uncertainty is found between the values (Table S3,† *K*_D_ (M^–1^): **1**: 450 (70), **2**: 240 (90), **3**: 600 (200)). This discrepancy is attributed to uncertainty found in the integrated spectral areas arising from errors in integration (Fig. S10–S12†) compounded by solvent subtraction (Fig. S8†). Regardless, further support of these results can be found by extrapolation to infinite dilution through eqn (4) as detailed by Luck (Fig. S9†).^49^
4
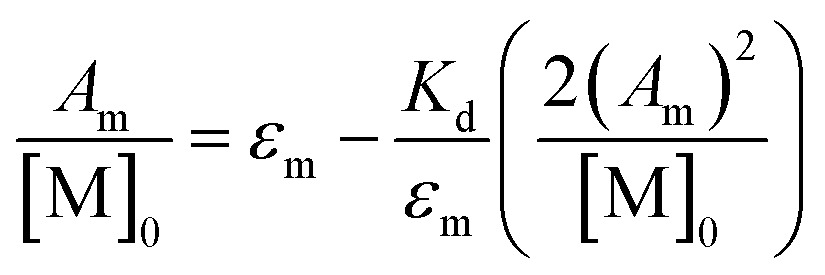



Here all values have their usual meanings, and excellent agreement is found upon comparison to those values determined by eqn (3) (Table S4,† *K*_D_ (M^–1^): **1**: 120 (7), **2**: 73 (5), **3**: 126 (9)). All three results support the notion that **1–3** form weak hydrogen bonds in solution at 25 °C (
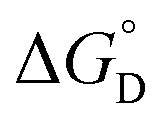
 (kcal mol^–1^), **1**: –2.83 (0.02), **2**: –2.56 (0.04), **3**: –2.88 (0.04)).

Previous ^1^H DOSY NMR experiments have shown that fully reduced solutions of **1–3** consist of hydrogen bonded dimers, supporting a *K*_2–_ ≥ 10^3^.^38^ These findings are confirmed through the determination of *K*_2–_ by UV/vis/NIR spectroscopy. Applying the same methodology for the determination of *K*_D_, the absorption spectra of (**1**_2_)^2–^, (**2**_2_)^2–^, (**3**_2_)^2–^, (Fig. S10–S12†) displays a broadened, intra-cluster-charge-transfer (ICCT) band in the visible region for both the anionic monomer, (**1**)^–^, (**2**)^–^, (**3**)^–^, and the dianionic hydrogen-bonded dimer, (**1**_2_)^2–^, (**2**_2_)^2–^, (**3**_2_)^2–^, species (Fig. S10–S12†). Upon comparison of the electronic spectra of similar, homoleptic clusters [Ru_3_(μ_3_-O)(OAc)_6_(CO)(L_1_)_2_]^–^ where L_1_ = cpy, py, or dmap (Fig. S13†) which are incapable of dimerizing, it is clear to see that the broadened ICCT band consists of both monomeric and dimeric contributions.^38,39,47,48,50^ *In lieu* of determining spectral areas, the peak heights of the monomeric band ((**1**)^–^: 612 nm, (**2**)^–^: 487 nm, (**3**)^–^: 550 nm) were used with eqn (2) and *K*_2–_ was found to range from 2000 to 2500 M^–1^ (Fig. S14;† Table 1, *K*_2–_ (M^–1^): (**1**)^–^: 2000 (400), (**2**)^–^: 2200 (300), (**3**)^–^: 2500 (300)). Unlike *K*_D_, *K*_2–_ was found to increase linearly with increasing electron-donating nature of the ancillary ligand (Fig. S15†). These values are further confirmed through eqn (4), where values are nearly identical within experimental error (Fig. S14; Table S6,† K_2–_ (M^–1^): (**1**)^–^: 2000 (400), (**2**)^–^: 2200 (300), (**3**)^–^: 2700 (300)) and indicate the formation of moderately strong hydrogen bonds in solution (
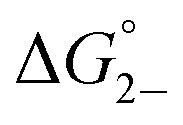
 (kcal mol^–1^), (**1**)^–^: –4.5 (0.1), (**2**)^–^: –4.56 (0.08), (**3**)^–^: –4.63 (0.07)).

The comproportionation constant (*K*_C_), is largely a measure of the thermodynamic stability of the mixed-valent (1–) state with respect to the isovalent states (0 and 2–) and can be determined from the electrochemical splitting (Δ*E*) of the 0/– and –/2– redox couples measured in a cyclic voltammogram (CV) through eqn (2).^51^
5
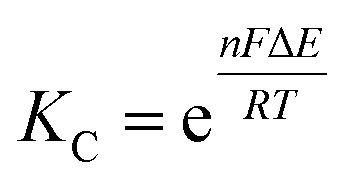



For complexes **1–3**, the values for *K*_C_ were determined from the electrochemical splittings of the return oxidation features observed in the cyclic voltammograms (CV, Fig. S16–S18†). At 23 °C in dichloromethane; for all clusters, *K*_C_ is found to be on the order of 10^6^ (**1**: *K*_C_ = 1.09 (0.04) × 10^6^, **2**: *K*_C_ = 3.2 (0.1) × 10^6^, **3**: *K*_C_ = 4.8 (0.2) × 10^6^) and increases with increasing p*K*_a_ of the ancillary pyridyl ligand.

**Fig. 3:**
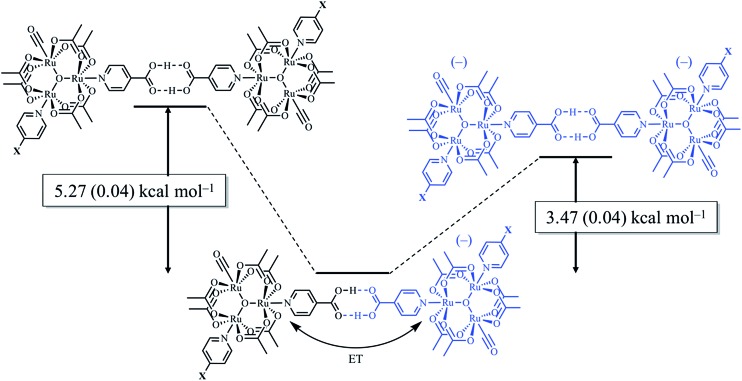
Relative free energy diagram of hydrogen-bond formation, showing the additional stabilization of hydrogen bonds participating in electron delocalization.

Utilizing eqn (1), *K*_MV_ was found to be on average four orders of magnitude larger than *K*_D_ and three orders of magnitude larger than *K*_2–_. *K*_MV_ was found to range from 0.5–1.2 × 10^6^ M^–1^ (Table 1) in DCM and increases linearly with increasing electron-donating nature of the ancillary pyridyl ligand (Fig. S15,† p*K*_a_: **1**, cpy = 1.9; **2**, py = 5.1; **3**, dmap = 9.2). This effect can be explained through a ligand-field description; as stronger donor ligands are used, the Ru_3_O d-manifold is raised into closer energetic alignment with the isonicotinic acid π* levels, giving rise to more resonant delocalization across the hydrogen bonded dimers.^52^ This description is consistent with the direct mixing of metal center and bridging ligand wave-functions providing an indirect method for donor–acceptor overlap.^39^ The difference in free energies obtained between *K*_MV,_ *K*_D_, and *K*_2–_ (ΔΔ*G*°, Table 1) reveal the relative stabilities of the mixed-valent states relative to the two isovalent hydrogen-bonded states. On average, a stabilization of –5.27 (0.04) kcal mol^–1^ (1850 (10) cm^–1^) and –3.47 (0.06) kcal mol^–1^ (1210 (20) cm^–1^) is gained upon the formation of mixed-valent, hydrogen-bonded dimers, (**1**_2_)^–^, (**2**_2_)^–^, (**3**_2_)^–^, relative to the neutral, **1**_2_, **2**_2_, **3**_2_ and dianionic, (**1**_2_)^2–^, (**2**_2_)^2–^, (**3**_2_)^2–^, states respectively (Fig. 3).

While the bonds joining the dimers of Ru_3_ clusters in the mixed valence states (**1**_2_)^–^, (**2**_2_)^–^, (**3**_2_)^–^ fulfill the definition of hydrogen bonds, their significantly larger than normal stabilities are derived from electron exchange. It is apparent that significant mixing of the metal and bridging ligand molecular orbitals in the mixed-valent states provide larger-than-expected electronic couplings for metal centers typically considered too far apart or too weakly interacting to show significant electronic interactions.^39^ To our knowledge, this is the first determination of the significant increase in the strength of hydrogen bonds when they participate in delocalization of an electron.

## Methods

### Preparation and purification

Complexes **1–3** were synthesized following previously reported procedures.^38,39^ The isonicotinic acid was used as received from MP Biomedical Inc. while the decamethyl ferrocene and decamethyl cobaltocene were used as received from Sigma-Aldrich. The cyclohexane stabilized dichloromethane (DCM), and tetrahydrofuran (THF) were purchased from VWR International LLC, deoxygenated and dried over alumina columns on a custom built solvent system under an argon atmosphere and stored over activated 4 Å molecular sieves in a nitrogen filled glove box.

### Chemical reductions

Stock solutions of 0.60 mM of **1–3** and 3.60 mM of Co(cp*)_2_ were prepared in dry THF under an inert atmosphere. From the stock solution of **1–3**, five aliquots were prepared for each sample directly into an air tight 10 mm path length quartz cuvette ranging in concentrations from 0.13 mM to 0.03 mM. The absorption spectrum of each aliquot was recorded prior to reduction to determine the exact molarity of each aliquot. A stoichiometric amount of Co(cp*)_2_ was then injected into each aliquot, using a Hamilton gas-tight microsyringe, to fully reduce the samples by one electron. After injection the cell was sealed and the absorption spectrum was promptly collected.

### Infrared data collection and analysis

Infrared spectra were collected on a Bruker Equinox 55 FTIR spectrometer using a SPECAC variable path length IR cell with CaF_2_ windows set to a path length of 2.0 mm. Solutions were prepared in a glove box under a nitrogen atmosphere using pre-dried DCM and subsequently analyzed. After solvent subtraction, *ν*(COOH) bands were fit to two, well resolved Gaussian functions using the Igor Pro software to obtain integrated spectral areas used in the equilibrium analysis. It is important to note that calculation of the dimerization constant should remain independent of band choice; however, discrepancy between the two values in this experiment (Table S3†) are attributed to errors in solvent subtraction resulting from a slight DCM absorbance between 1730 and 1700 cm^–1^ coinciding with the dimeric *ν*(COOH) stretch at 1711 cm^–1^ (Fig. S8†).

### UV/visible data collection and analysis

UV-visible spectra were collected on a Shimadzu UV-3600 UV/vis/NIR spectrometer. Samples for determination of *K*_2–_ were diluted directly into air tight 10 mm path length quartz cuvettes from stock solutions of 1–3. Samples from the determination of *K*_D_ were taken directly from FTIR solutions and enclosed in a 1.0 mm path length, Hellma Analytics QS® high precision cell.

### Electrochemical measurements

Electrochemistry was performed on a BASi Epsilon potentiostat, in dried degassed DCM with 0.1 M tetrabutylammonium hexafluorophosphate (TBAPF_6_, recrystallized from MeOH vacuum dried at 80 °C) used as a supporting electrolyte. Cyclic voltammograms (CVs) and differential pulse voltammograms (DPVs) were recorded at 298 K with ∼2.7 mM analyte concentrations using a three electrode setup consisting of a glassy carbon working electrode (3 mm diameter), a Pt auxiliary electrode, and an Ag/AgCl wire reference electrode. All samples were referenced to the ferrocene +/0 redox couple using an internal standard of decamethyl ferrocene (*E*_1/2_ = –0.59 *vs.* Fc^+/0^).

## Author contributions

T. M. P. designed and performed the experiments. G. P. H. aided in data collection and synthesis. C. P. K oversaw the project. All authors analyzed the data and prepared the manuscript.

## Conflicts of interest

There are no conflicts to declare.
