# Supplementary material for: Effects of electron transfer on the stability of hydrogen bonds
Source: Chem Sci. 2017 Aug 30;8(11):7324–9. doi: 10.1039/c7sc03361c (PMC5672789; doi:10.1039/c7sc03361c)
Supplement: Supplementary file 1 [file SC-008-C7SC03361C-s001.pdf]

Electronic Supplementary Material (ESI) for Chemical Science.
This journal is © The Royal Society of Chemistry 2017

Supplementary Materials for

Effects of Electron Transfer on the Stability of Hydrogen Bonds

Tyler M. Porter, Gavin P. Heim, and Clifford P. Kubiak*.

correspondence to: ckubiak@ucsd.edu

This PDF file includes:

Materials and Methods
Supplementary Text
Figs. S1 to S22
Tables S1 to S6

Supplementary Text

Derivation of K_{MV}

$$M + M \rightleftharpoons M_2 \quad K_D$$
$$M + (M)^- \rightleftharpoons (M)_2^- \quad K_{MV}$$
$$(M)^- + (M)^- \rightleftharpoons (M)_2^{-2} \quad K_{-2}$$
$$M_2 + (M)_2^{-2} \rightleftharpoons 2\,(M)_2^- \quad K_C$$

$$[1]^2 = \frac{[(1)_2]}{K_D} \qquad \left[(1)_2^-\right] = K_C\left[(1)_2^{2-}\right][(1)_2] \qquad \left[(1)^-\right] = \frac{\left[(1)_2^{2-}\right]}{K_{2-}}$$

$$(K_{mv})^2 = \frac{K_C\left[(1)_2^{2-}\right][(1)_2]}{\left(\frac{[(1)_2]}{K_D}\right)\left(\frac{\left[(1)_2^{2-}\right]}{K_{2-}}\right)}$$

$$(K_{mv})^2 = K_C K_{2-} K_D$$

$$K_{mv} = (K_C K_{2-} K_D)^{1/2}$$

Derivation of Dimerization Equations

Consider a 1:1 self-dimerization:

$M + M \rightleftharpoons M_2$

$$K_D = \frac{[M_2]}{[M]^2} \qquad [M]_0 = [M] + 2[M_2]$$

Solving the mass balance for $[M_2]$ and substituting into K_D

$$K_D = \frac{[M]_0 - [M]}{2[M]^2}$$

Using the quadratic equation to solve for [M] and keeping the positive solution yields:

$$[M] = \frac{-1 + (1 + 8K_D[M]_0)^{1/2}}{4K_D}$$

Multiplying top and bottom by the conjugate of $(-1 - (1 + 8K_D[M]_0)^{\frac{1}{2}})$ yields:

$$[M] = \frac{2[M]_0}{1 + (1 + 8K_D[M]_0)^{1/2}}$$

Substitution of [M] into mass balance equation and solving for $[M_2]$ yields:

$$[M_2] = \frac{[M]_0}{2} - \frac{2[M]_0}{1 + (1 + 8K_D[M]_0)^{1/2}}$$

Combine using a common denominator of $2(1 + (1 + 8K_D[M]_0)^{\frac{1}{2}})$

$$[M_2] = \frac{[M]_0\left(1 + (1 + 8K_D[M]_0)^{\frac{1}{2}}\right) - 2[M]_0}{2\left(1 + (1 + 8K_D[M]_0)^{\frac{1}{2}}\right)}$$

$$[M_2] = \left(\frac{[M]_0}{2}\right)\left(\frac{(1 + 8K_D[M]_0)^{\frac{1}{2}} - 1}{(1 + 8K_D[M]_0)^{\frac{1}{2}} + 1}\right)$$

From the above we end at two expressions for the monomer and dimer concentrations in solution.

$$[M] = \frac{2[M]_0}{1 + (1 + 8K_D[M]_0)^{1/2}}$$

$$[M_2] = \left(\frac{[M]_0}{2}\right)\left(\frac{(1 + 8K_D[M]_0)^{\frac{1}{2}} - 1}{(1 + 8K_D[M]_0)^{\frac{1}{2}} + 1}\right)$$

Using the Beer-Lambert law we can then describe the absorbance of monomer and dimer bands as follows:

$$A_m(\tilde{\nu}) = \varepsilon_m(\tilde{\nu})l[M] \qquad A_d(\tilde{\nu}) = \varepsilon_d(\tilde{\nu})l[M_2]$$

Where $A_m(\tilde{\nu})$ and $A_d(\tilde{\nu})$ are the absorbance of the monomer and dimer at a specific wavelength, $\varepsilon_m(\tilde{\nu})$ and $\varepsilon_m(\tilde{\nu})$ are the molar absorptivity at $\tilde{\nu}$, and l the optical path length. The integrated absorbance of the whole band is then given by:

$$A_m = (l[M])\int \varepsilon_m(\tilde{\nu})d\tilde{\nu} \qquad A_d = (l[M_2])\int \varepsilon_d(\tilde{\nu})d\tilde{\nu}$$

Where A_m and A_d are now the integrated absorbance's of the monomer and dimer bands respectively and $\int \varepsilon_m(\tilde{\nu})d\tilde{\nu}$ and $\int \varepsilon_d(\tilde{\nu})d\tilde{\nu}$ are the molar absorptivity's of the monomer and dimer bands respectively. Substituting in for the concentrations of [M] and $[M_2]$ then yields:

$$A_m = \frac{2[M]_0 \varepsilon_m l}{1 + (1 + 8K_D[M]_0)^{1/2}}$$

$$A_d = \left(\frac{\varepsilon_d l[M]_0}{2}\right)\left(\frac{(1 + 8K_D[M]_0)^{\frac{1}{2}} - 1}{(1 + 8K_D[M]_0)^{\frac{1}{2}} + 1}\right)$$

Focusing on the monomer equation, we can divide the first and last terms by $2[M]_0 \varepsilon_m l$ and inverting the resulting fraction yields:

$$\frac{2[M]_0 \varepsilon_m l}{A_m} = 1 + (1 + 8K_D[M]_0)^{1/2}$$

If we set $a = \frac{2[M]_0 \varepsilon_m l}{A_m}$, subtract by unity, take the squares of both sides, and simplify we arrive at:

$$(a - 1)^2 = 1 + 8K_D[M]_0$$

$$a^2 - 2a + 1 = 1 + 8K_D[M]_0$$

$$a^2 = 2a + 8K_D[M]_0$$

$$\left(\frac{2[M]_0 \varepsilon_m l}{A_m}\right)^2 = \frac{4[M]_0 \varepsilon_m l}{A_m} + 8K_D[M]_0$$

We can then linearize by dividing both sides by $\left(\frac{4{\varepsilon_m}^2 l^2[M]_0}{A_m}\right)$

$$\left(\frac{A_m}{4{\varepsilon_m}^2 l^2[M]_0}\right)\left(\frac{2[M]_0 \varepsilon_m l}{A_m}\right)^2 = \left(\frac{4[M]_0 \varepsilon_m l}{A_m} + 8K_D[M]_0\right)\left(\frac{A_m}{4{\varepsilon_m}^2 l^2[M]_0}\right)$$

$$\frac{[M]_0}{A_m} = \left(\frac{2K_D}{{\varepsilon_m}^2 l^2}\right)A_m + \frac{1}{\varepsilon_m l}$$

We can treat the dimer band in the same manner, remembering that:

$$A_d = \left(\frac{\varepsilon_d l[M]_0}{2}\right)\left(\frac{(1 + 8K_D[M]_0)^{\frac{1}{2}} - 1}{(1 + 8K_D[M]_0)^{\frac{1}{2}} + 1}\right)$$

Divide both sides of the equation by $\left(\frac{\varepsilon_d l[M]_0}{2}\right)$:

$$\frac{2A_d}{\varepsilon_d l[M]_0} = \frac{(1+8K_D[M]_0)^{\frac{1}{2}} - 1}{(1+8K_D[M]_0)^{\frac{1}{2}} + 1}$$

Using the relationship if $\frac{a}{b} = \frac{c}{d}$ then $\frac{a+b}{a-b} = \frac{c+d}{c-d}$ is we can reduce to the following:

$$\frac{\varepsilon_d l[M]_0 + 2A_d}{\varepsilon_d l[M]_0 - 2A_d} = (1+8K_D[M]_0)^{\frac{1}{2}}$$

$$a = \varepsilon_d l[M]_0 \qquad b = 2A_d$$

$$c = (1+8K_D[M]_0)^{\frac{1}{2}} \qquad d = 1$$

Again using the relationship that if $\frac{a}{b} = \frac{c}{d}$ then $\frac{a-b}{b} = \frac{c-d}{d}$ we arrive at:

$$\frac{\varepsilon_d l A_d}{K_D} = (\varepsilon_d l[M]_0 - 2A_d)^2$$

We can then linearize the expression by taking the square roots of both sides and dividing by $[M]_0$ we get:

$$\frac{{\varepsilon_d}^{1/2} l^{1/2} {A_d}^{1/2}}{[M]_0 {K_D}^{1/2}} = \varepsilon_d l - \frac{2A_d}{[M]_0}$$

$$\frac{2A_d}{[M]_0} = \varepsilon_d l - \left(\frac{\varepsilon_d l}{K_D}\right)^{1/2} \left(\frac{A_d^{\frac{1}{2}}}{[M]_0}\right)$$

We now have two linear expressions for the determination of K_D from both the monomer band and the dimer band.

$$\frac{[M]_0}{A_m} = \left(\frac{2K_D}{{\varepsilon_m}^2 l^2}\right) A_m + \frac{1}{\varepsilon_m l}$$

$$\frac{2A_d}{[M]_0} = \varepsilon_d l - \left(\frac{\varepsilon_d l}{K_D}\right)^{1/2} \left(\frac{A_d^{\frac{1}{2}}}{[M]_0}\right)$$

Where now a plot of $\frac{[M]_0}{A_m}$ vs. A_m would yield a line with a slope $p = \left(\frac{2K_D}{{\varepsilon_m}^2 l^2}\right)$ and an intercept of $q = \frac{1}{\varepsilon_m l}$ where K_D can then be found by:

$$p = \frac{2K_D}{{\varepsilon_m}^2 l^2} \qquad q = \frac{1}{\varepsilon_m l}$$

$K_D = \frac{p}{2q^2}$

$\varepsilon_m = \left(\frac{1}{q}\right)\left(\frac{1}{l}\right)$

While a plot of $\frac{2A_d}{[M]_0}$ vs. $\frac{A_d^{\frac{1}{2}}}{[M]_0}$ would yield a line with a slope $p = -\left(\frac{\varepsilon_d l}{K_D}\right)^{1/2}$ and intercept $q = \varepsilon_m l$ where K_D could then be found by:

$$p = -\left(\frac{\varepsilon_d l}{K_D}\right)^{\frac{1}{2}} \qquad q = \varepsilon_m l$$

$K_D = \frac{q}{p^2}$

$\varepsilon_m = \frac{q}{l}$

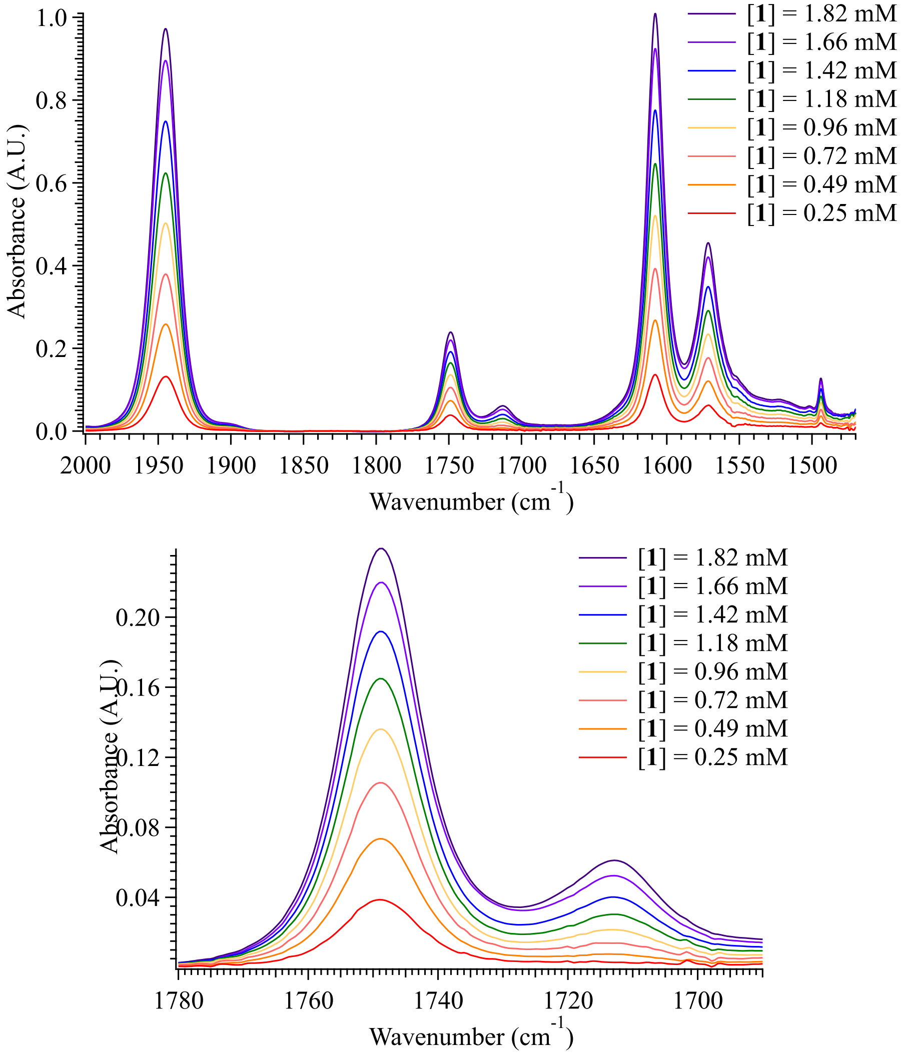

Fig. S1. (top) FTIR Spectrum of **1** in DCM at 25 °C. (bottom) ν(*CO*OH) stretching region.

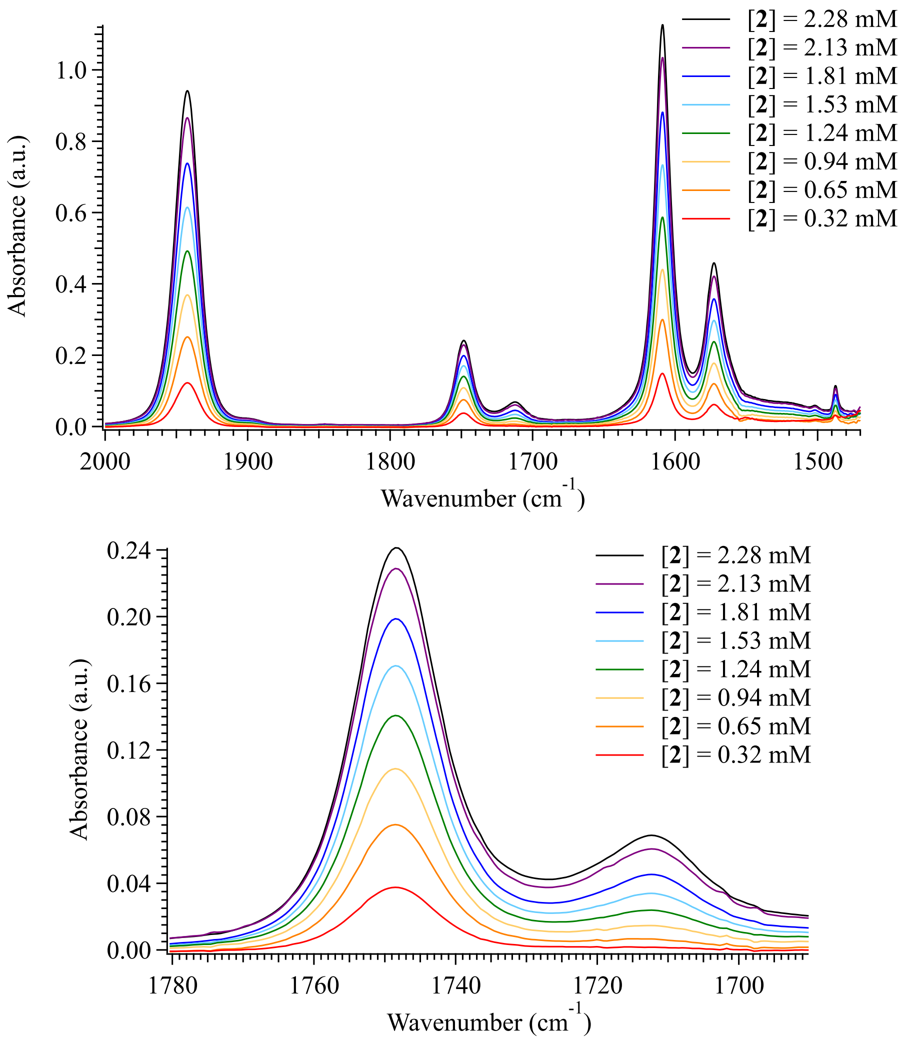

Fig. S2. (top) FTIR Spectrum of **2** in DCM at 25 °C. (bottom) ν(*CO*OH) stretching region.

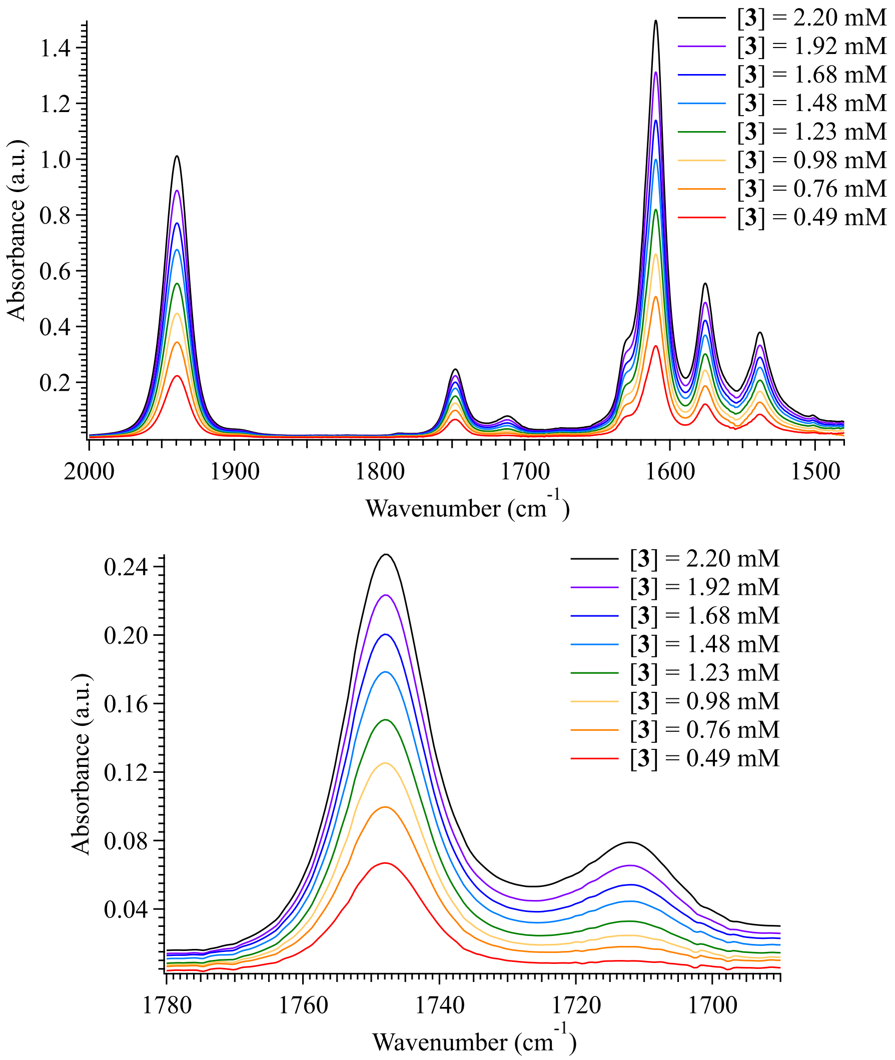

Fig. S3. (top) FTIR Spectrum of **3** in DCM at 25 °C. (bottom) ν(*CO*OH) stretching region.

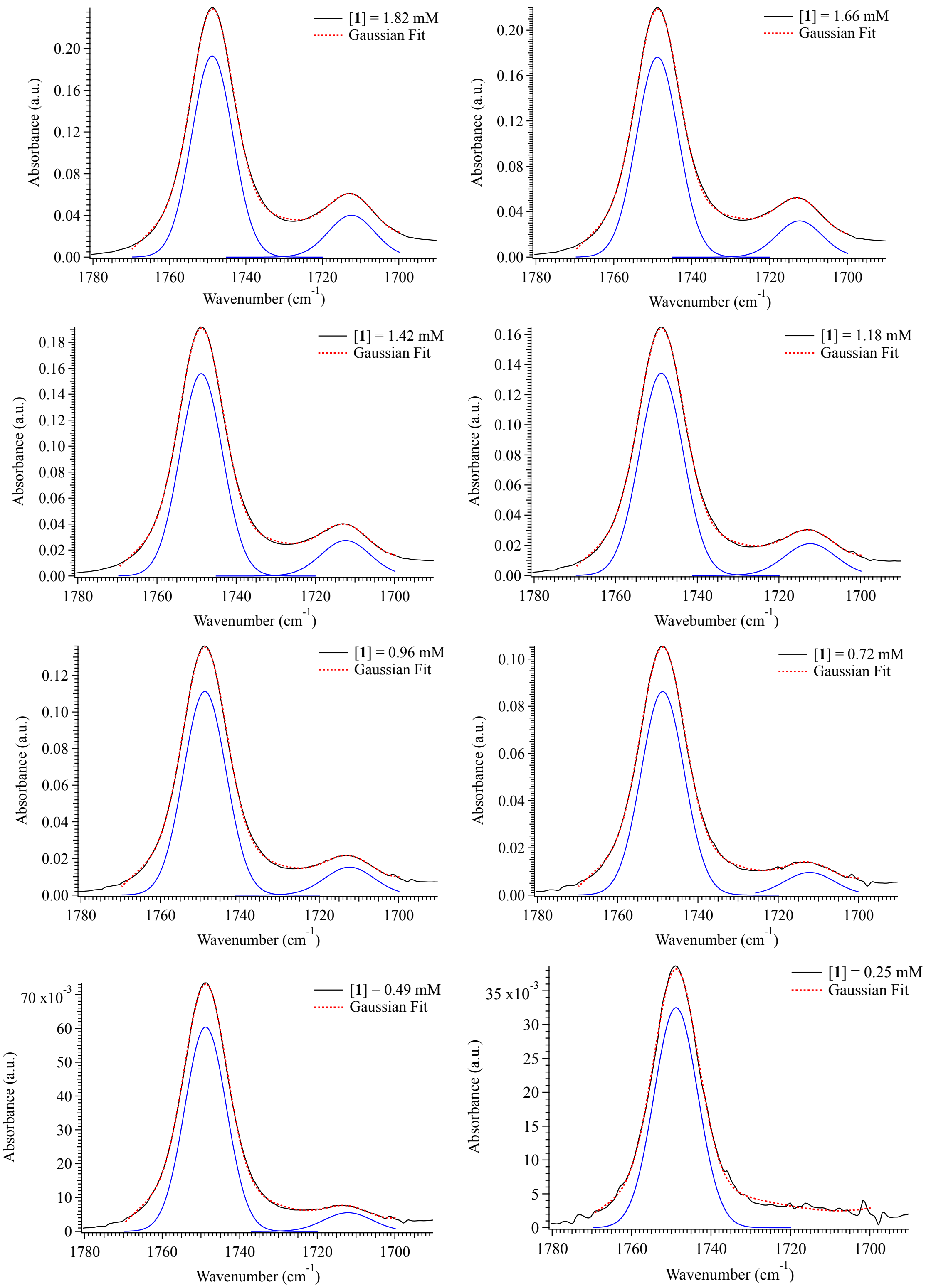

Fig. S4. FTIR Gaussian fits of ν(*CO*OH) stretching region of **1** in DCM.

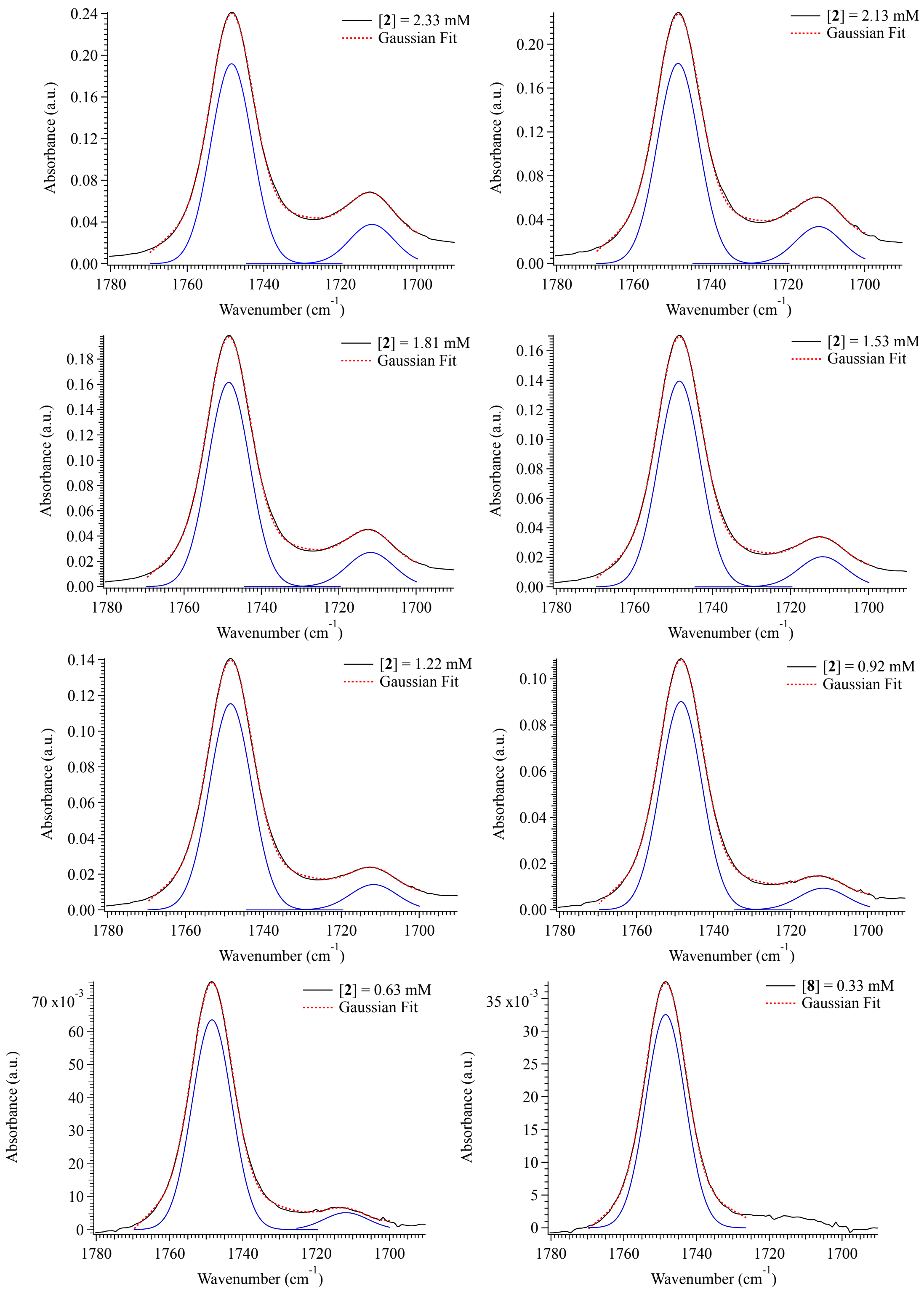

Fig. S5. FTIR Gaussian fits of ν(*CO*OH) stretching region of **2** in DCM.

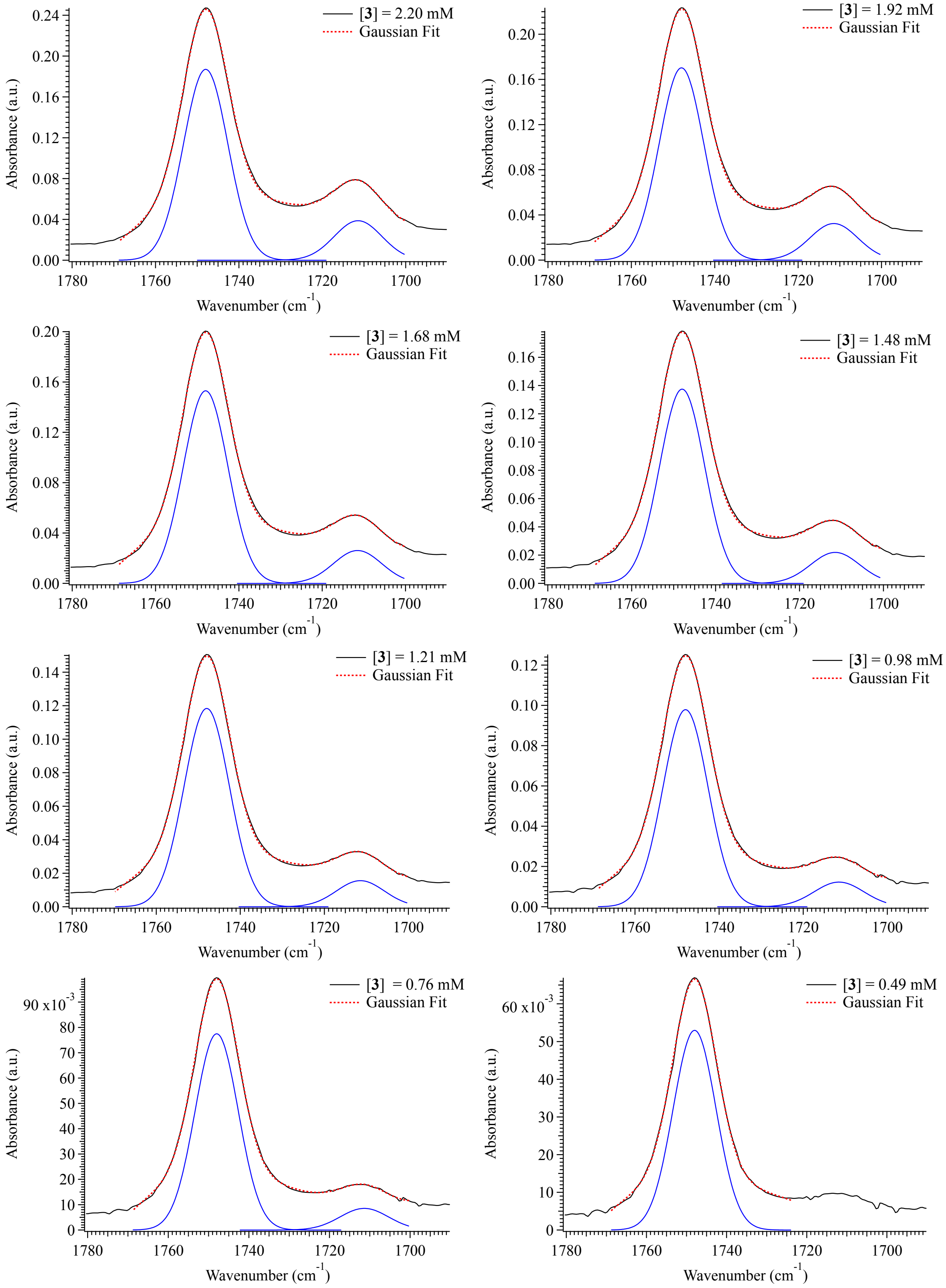

Fig. S6. FTIR Gaussian fits of ν(*CO*OH) stretching region of **3** in DCM.

$$\frac{[M]_0}{A_m} = \frac{1}{\varepsilon_m l} + \left(\frac{2K_d}{(\varepsilon_m l)^2}\right)A_m$$

$$\frac{2A_d}{[M]_0} = \varepsilon_d l - \left(\frac{\varepsilon_d l}{K_d}\right)^{1/2}\frac{(A_d)^{1/2}}{[M]_0}$$

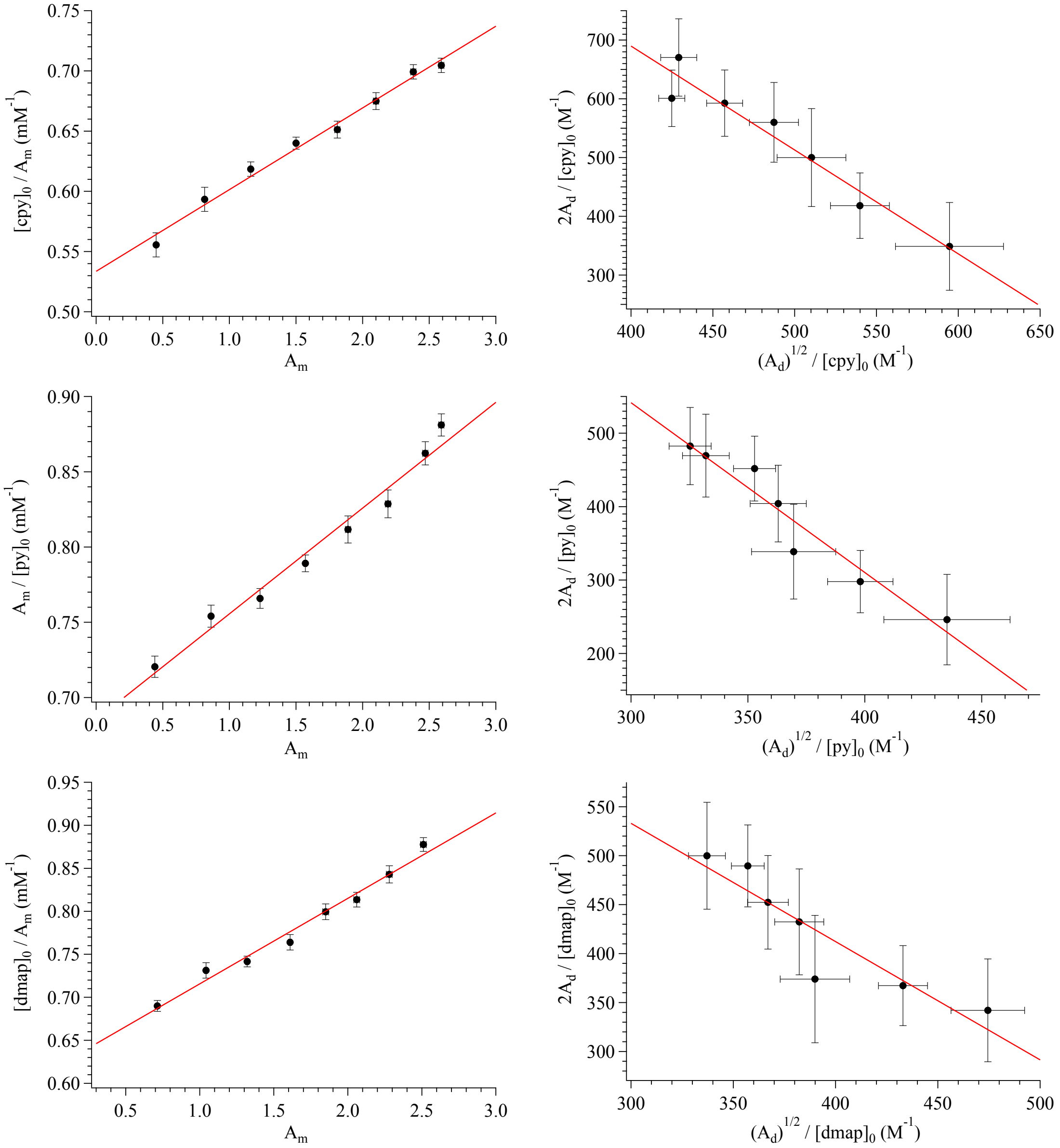

Fig. S7. Linear regression of integrated spectral areas for **1** (top), **2** (middle) **3** (bottom). Left side represents monomeric band while right side represents dimer band. $[M]_0$ is the stoichiometric concentration of the solute, A_m and A_d are the integrated spectral areas of the monomeric and dimeric bands respectively, ε_m and ε_d are the molar absorbtivities of the monomeric and dimeric bands respectively, l is the cell path lengths.

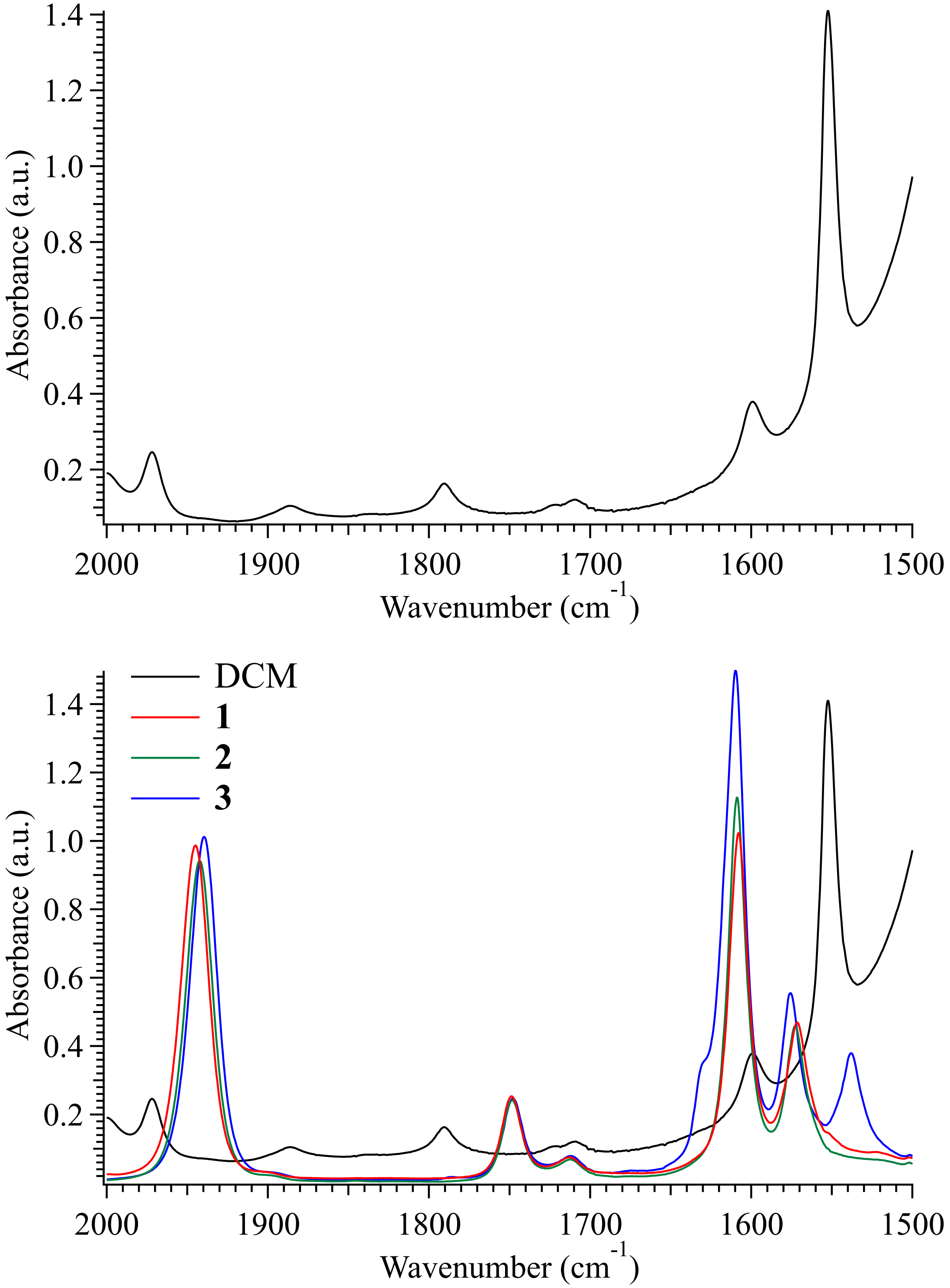

Fig. S8. (top) FTIR spectrum of DCM at a path length of 2.0 mm. (bottom) Overlaid FTIR spectrum of DCM and solvent subtracted **1**–**3**, noting the overlapping absorbance's between 1730 and 1700 cm^{-1}.

$$\frac{A_m}{[M]_0} = \varepsilon_m - \frac{K_d}{\varepsilon_m}\left(\frac{(2A_m)^2}{[M]_0}\right)$$

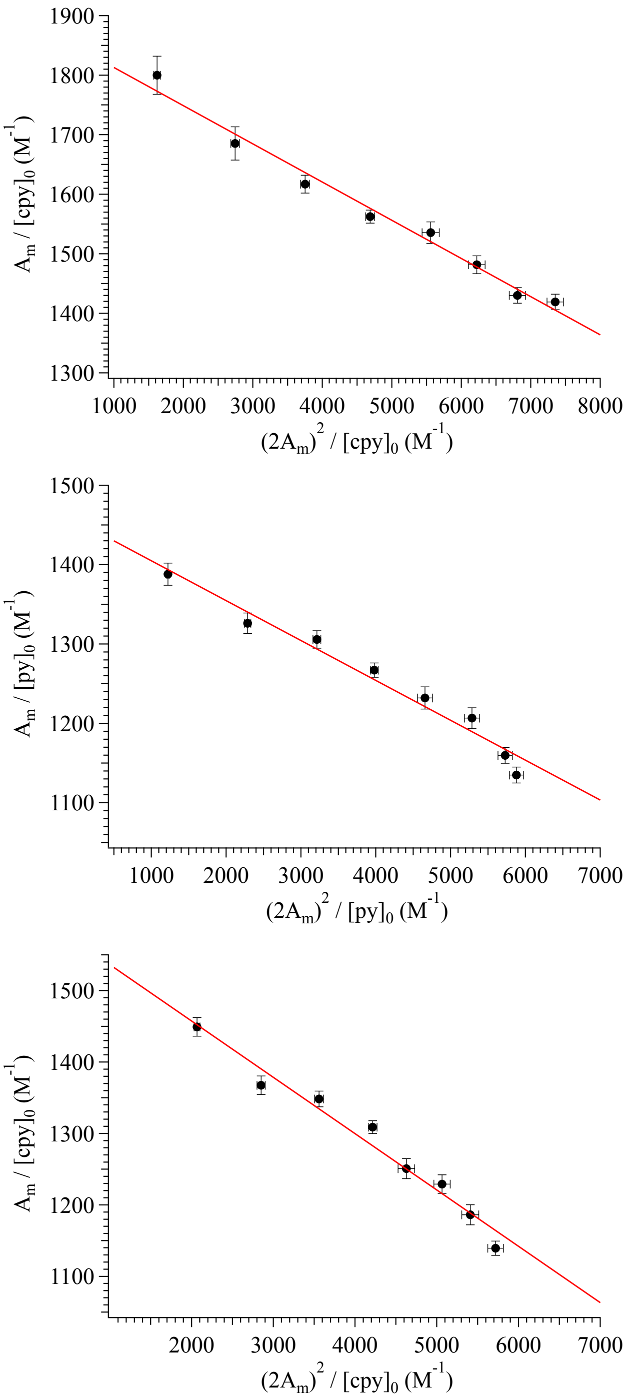

Fig. S9. Linear regression of integrated spectral areas for **1** (top), **2** (middle) **3** (bottom). Left side represents monomeric band while right side represents dimer band. $[M]_0$ is the stoichiometric concentration of the solute, A_m is the integrated spectral area of the monomer band, and ε_m is the extinction coefficient of the monomer band.

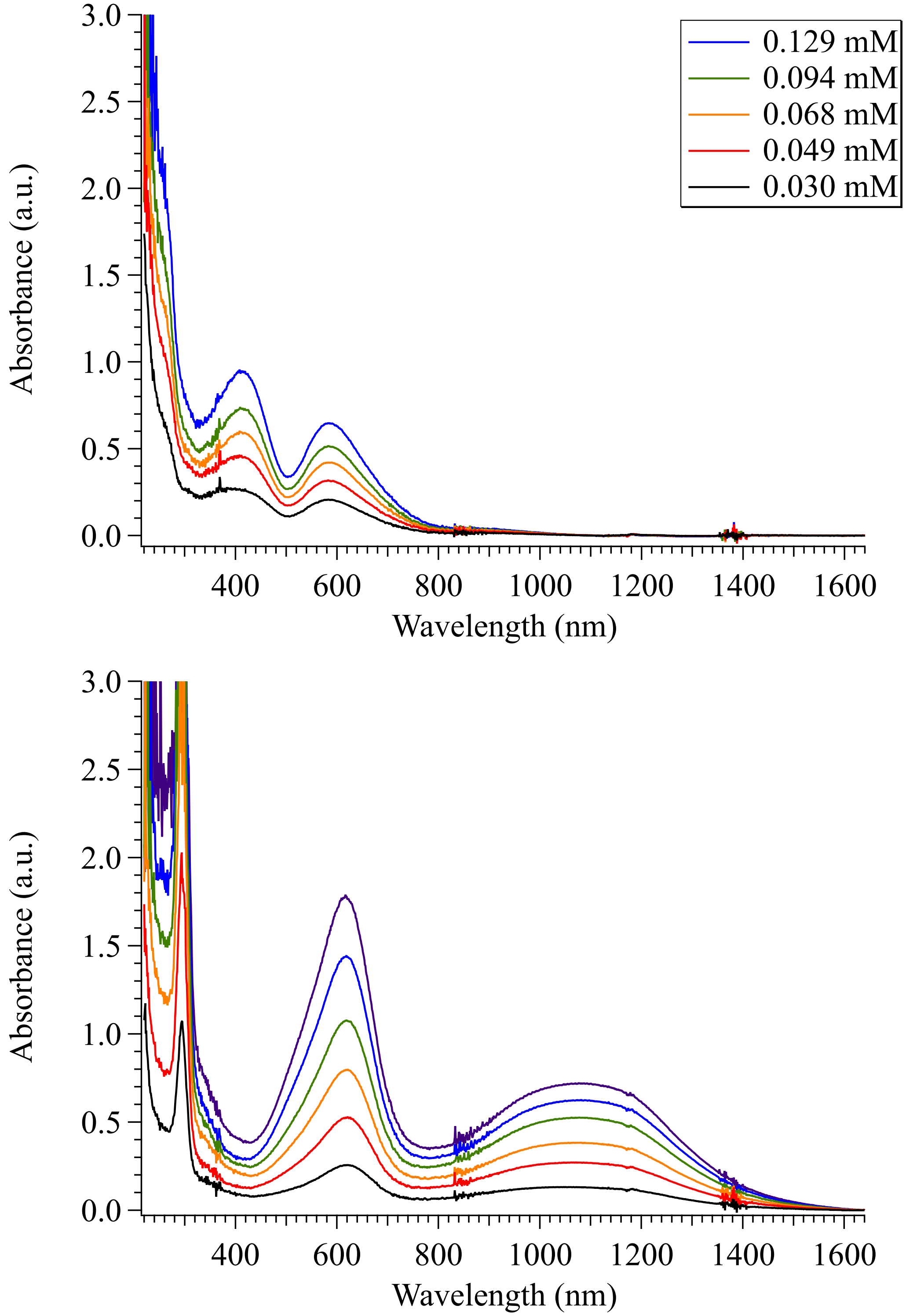

Fig. S10. (top) UV/vis/NIR spectra of **1** in THF. (bottom) UV/vis/NIR spectra $(\mathbf{1}_2)^{2-}$ in THF with $Co(cp^*)_2$ as a chemical reductant.

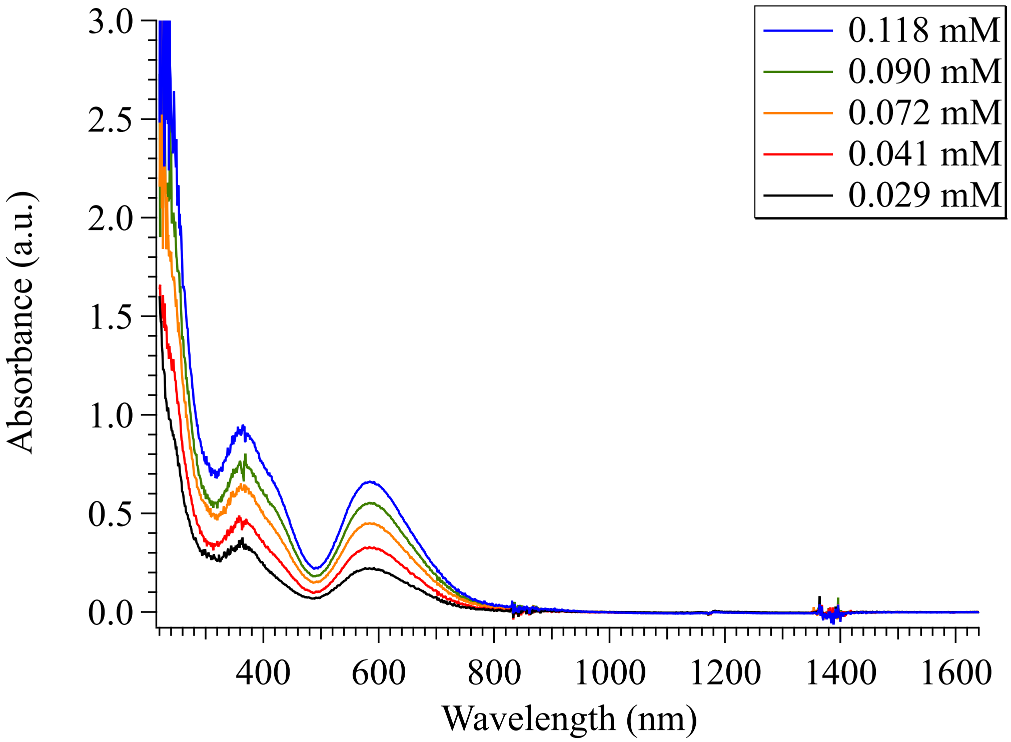

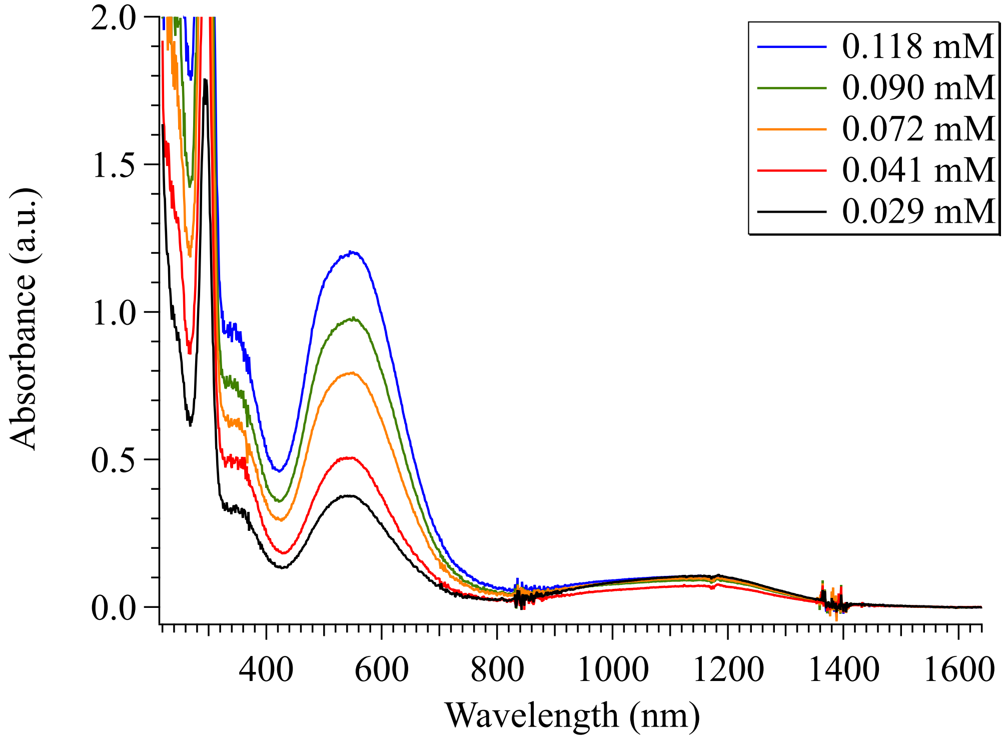

Fig. S11. (top) UV/vis/NIR spectra of **2** in THF. (bottom) UV/vis/NIR spectra $(\mathbf{2}_2)^{2-}$ in THF with $Co(cp^*)_2$ as a chemical reductant.

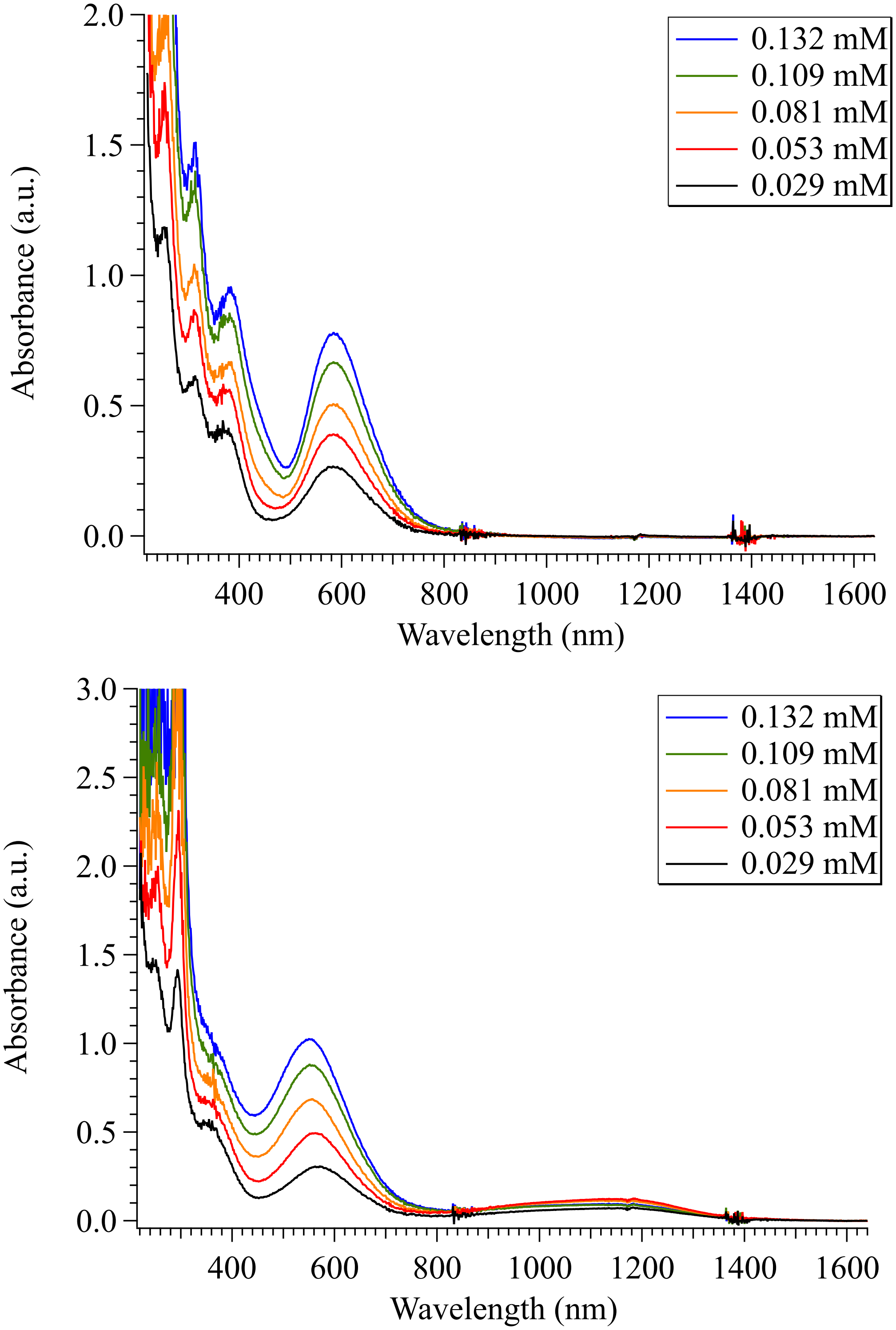

Fig. S12. (top) UV/vis/NIR spectra of **3** in THF. (bottom) UV/vis/NIR spectra $(\mathbf{3}_2)^{2-}$ in THF with $Co(cp^*)_2$ as a chemical reductant.

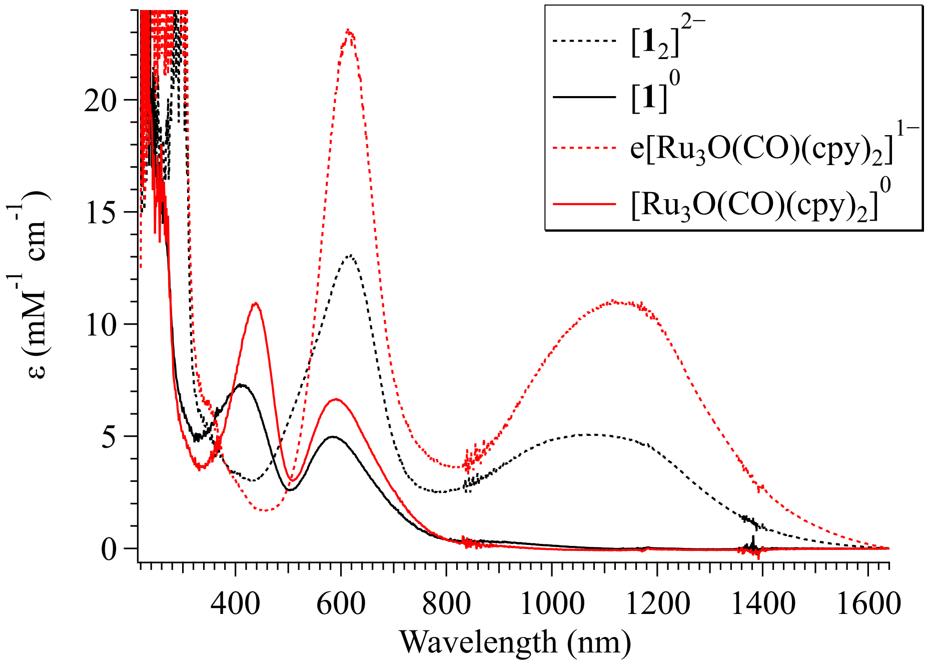

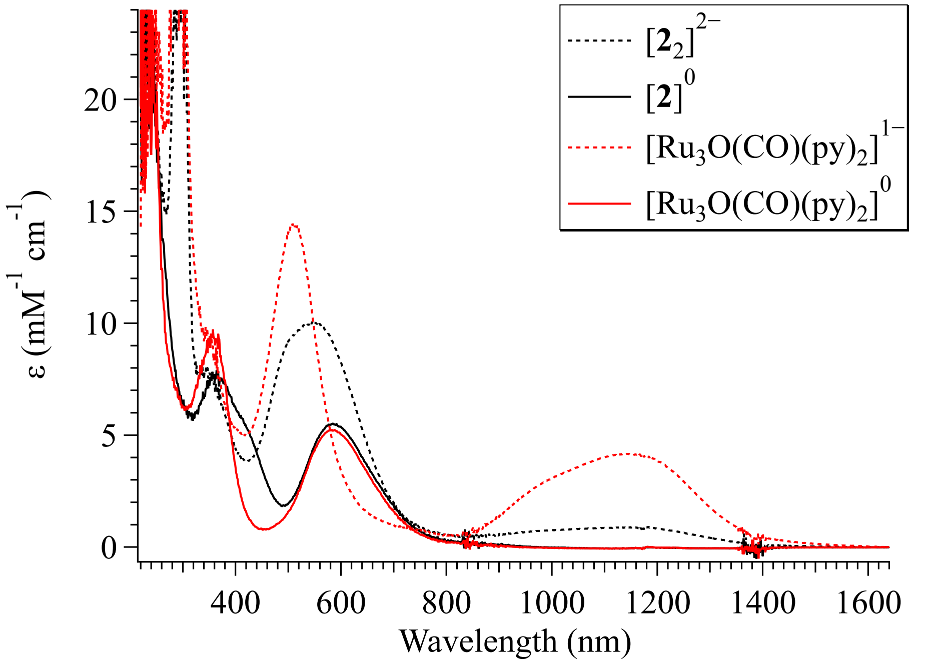

Fig. S13. (top) UV/vis/NIR of **1** and $(\mathbf{1}_2)^{2-}$ in black, and $[Ru_3O(OAc)_6(CO)(cpy)_2]$ and $[Ru_3O(OAc)_6(CO)(cpy)_2]^{-}$ in red in THF at 25 °C with $Co(cp^*)_2$ as a chemical reductant. (bottom) UV/vis/NIR of **2** and $(\mathbf{2}_2)^{2-}$ in black, and $[Ru_3O(OAc)_6(CO)(py)_2]$ and $[Ru_3O(OAc)_6(CO)(py)_2]^{-}$ in red in THF at 25 °C with $Co(cp^*)_2$ as a chemical reductant.

$$\frac{[M]_0}{H_m} = \frac{1}{\varepsilon_m l} + \left(\frac{2K_d}{(\varepsilon_m l)^2}\right) H_m \qquad \frac{A_m}{[M]_0} = \varepsilon_m - \frac{K_d}{\varepsilon_m}\left(\frac{(2A_m)^2}{[M]_0}\right)$$

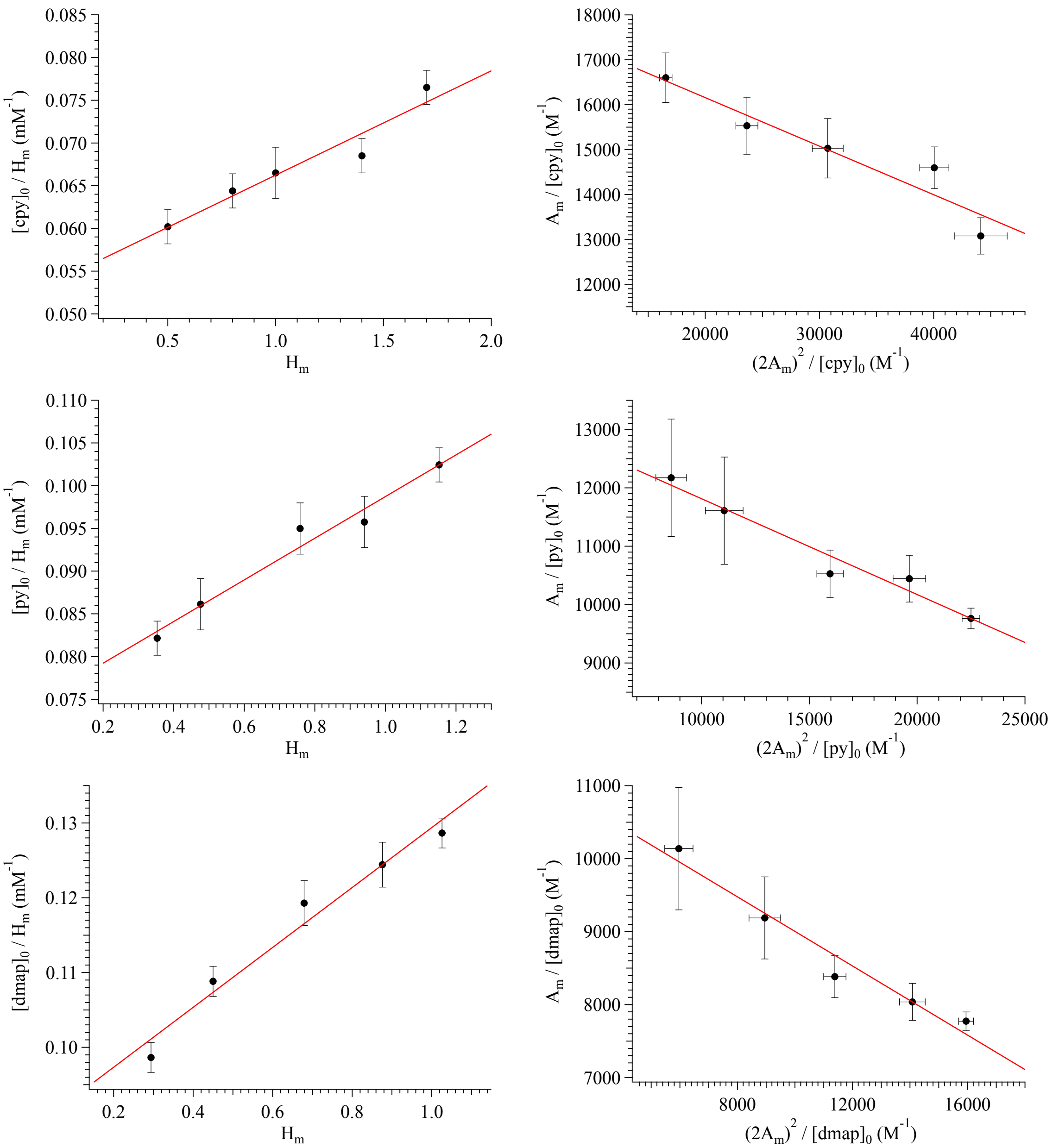

Fig. S14. (left) Linear regression of spectral heights for $(\mathbf{1}_2)^{2-}$ (top), $(\mathbf{2}_2)^{2-}$ (middle) $(\mathbf{3}_2)^{2-}$ (bottom) following equation 2. (right) Linear regression of spectral heights for $(\mathbf{1}_2)^{2-}$ (top), $(\mathbf{2}_2)^{2-}$ (middle) $(\mathbf{3}_2)^{2-}$ (bottom) following equation 4. $[M]_0$ is the stoichiometric concentration of the solute, H_m is the peak height of the monomer band, ε_m is the molar absorbtivity of the monomer bands, and l is the cell path length.

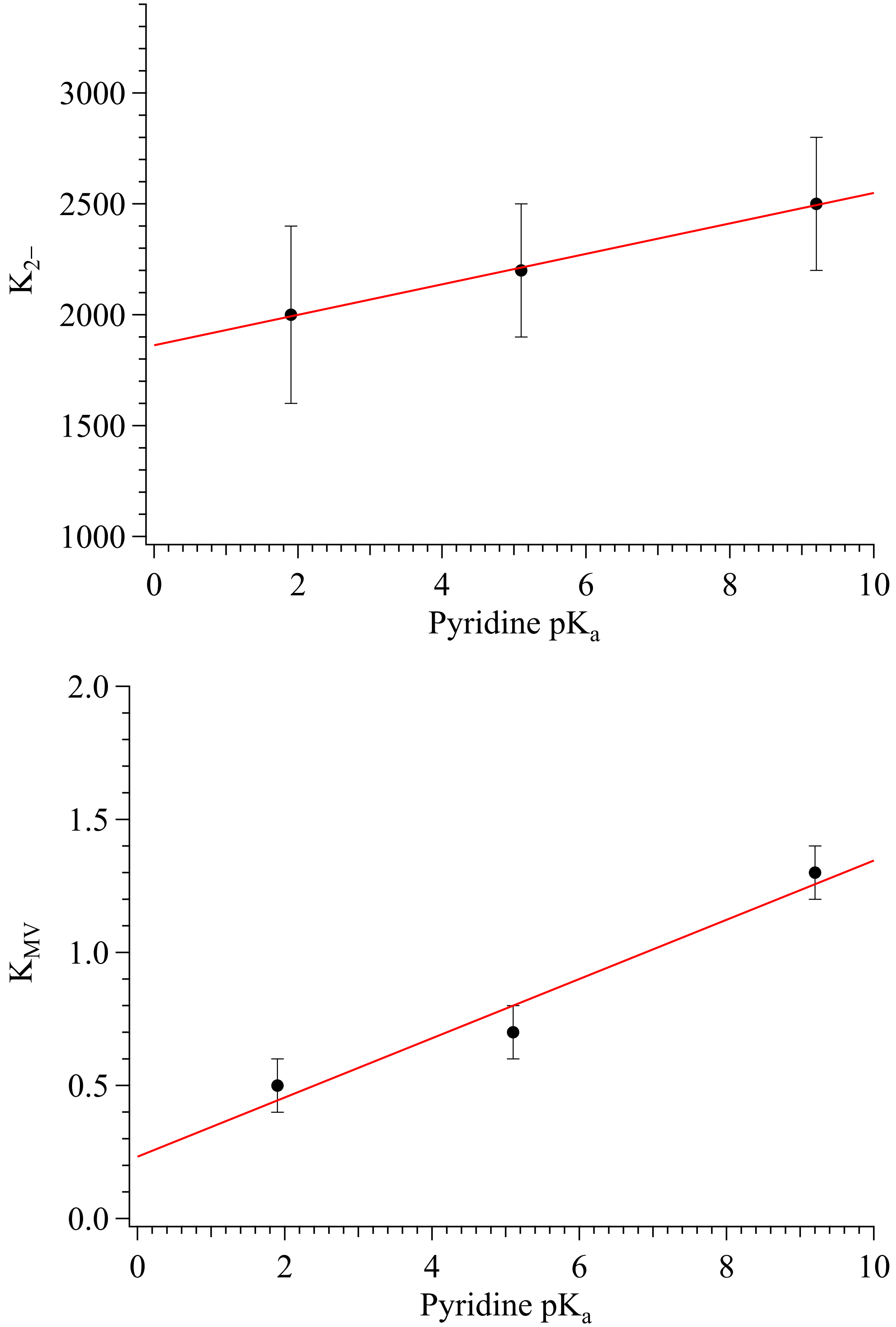

Fig. S15. (top) Plot of K_{2-} vs. pyridine pK_a. (bottom) Plot of K_{MV} vs. pyridine pK_a.

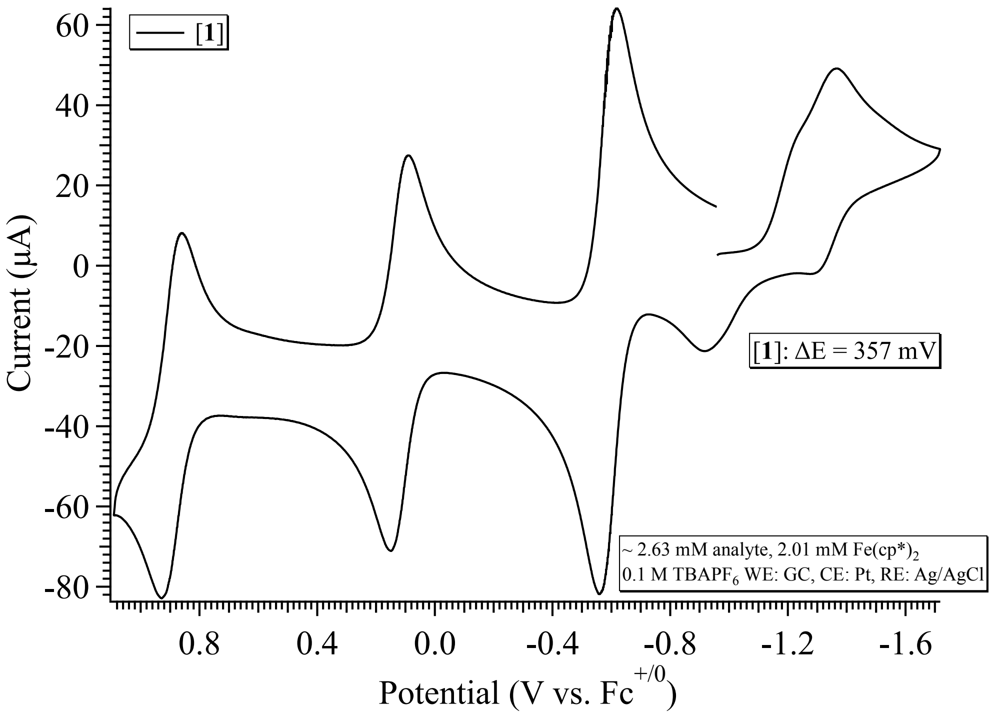

Fig. S16. Cyclic voltammogram of **1** in DCM at analyte concentrations of 2.63 mM with 0.1 M $TBAPF_6$ and an internal standard of decamethyl ferrocene ($E_{1/2} = -0.58$ V vs. $Fc^{+/0}$). CV's were recorded at 100 mV/s and referenced to the ferrocene +/0 redox couple.

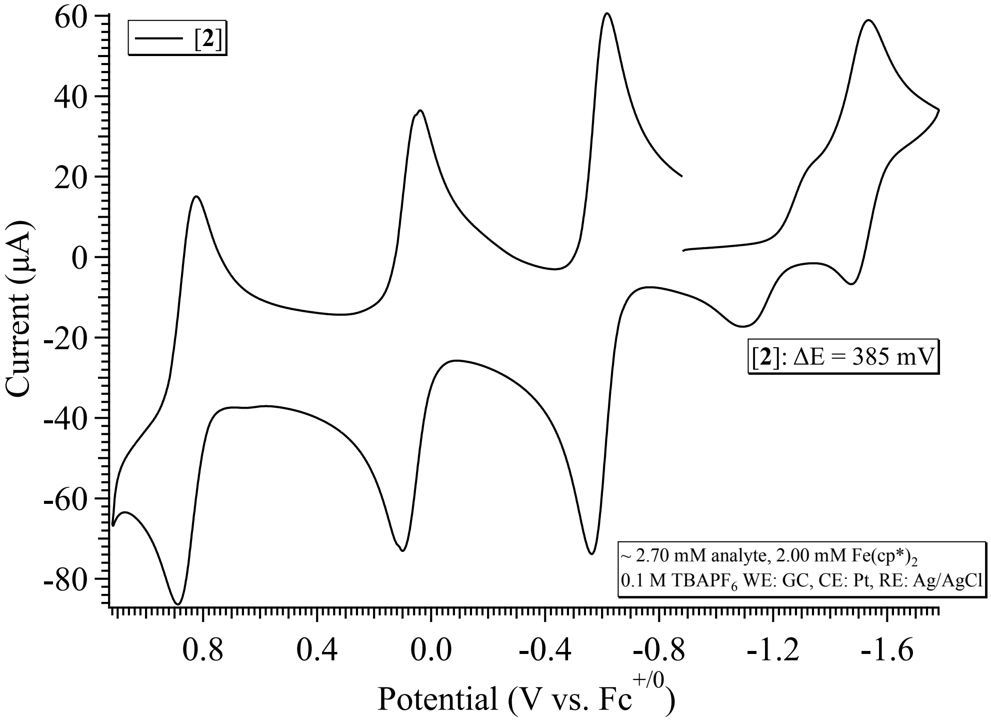

Fig. S17. Cyclic voltammogram of **2** in DCM at analyte concentrations of 2.70 mM with 0.1 M $TBAPF_6$ and an internal standard of decamethyl ferrocene ($E_{1/2} = -0.58$ V vs. $Fc^{+/0}$). CV's were recorded at 100 mV/s and referenced to the ferrocene +/0 redox couple.

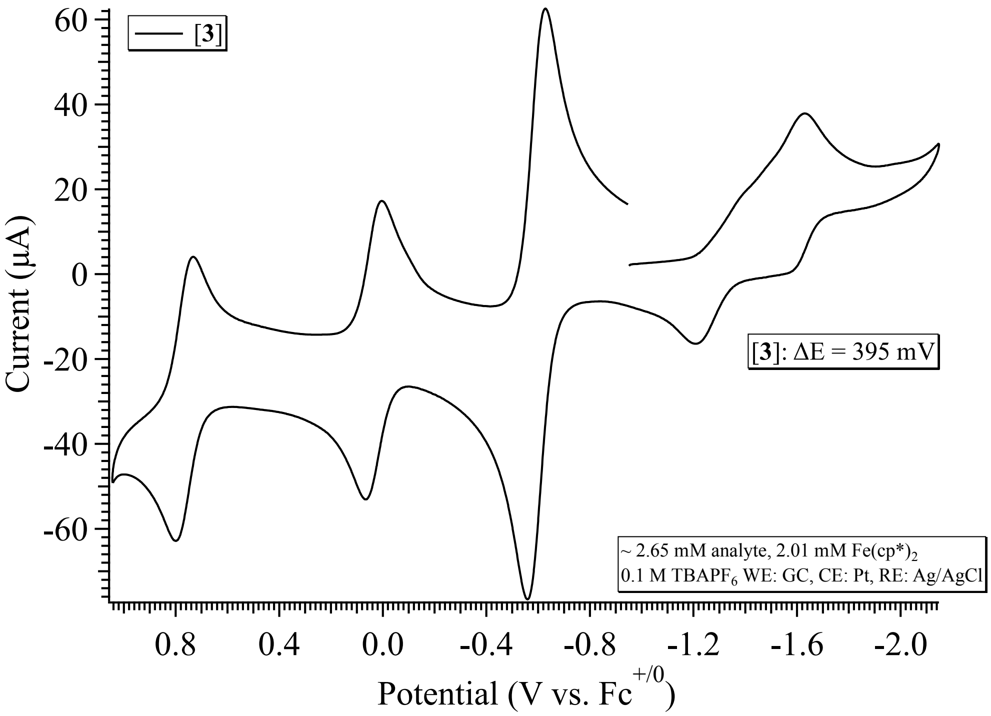

Fig. S18. Cyclic voltammogram of 3 in DCM at analyte concentrations of 2.65 mM with 0.1 M $TBAPF_6$ and an internal standard of decamethyl ferrocene ($E_{1/2}$ = –0.58 V vs. $Fc^{+/0}$). CV's were recorded at 100 mV/s and referenced to the ferrocene +/0 redox couple.

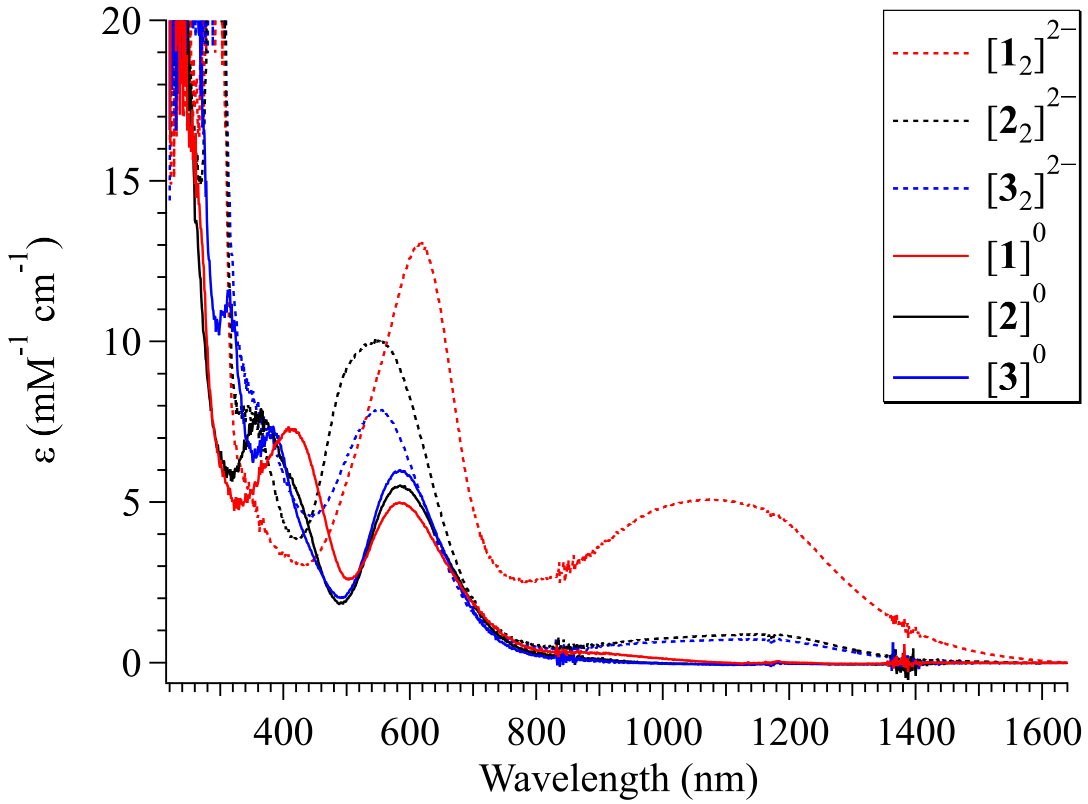

Fig. S19. UV/vis/NIR of **1**–**3** (solid) and $(\mathbf{1_2})^{2-}$–$(\mathbf{3_2})^{2-}$ (dashed) in THF at 25 °C with $Co(cp^*)_2$ used as a chemical reductant.

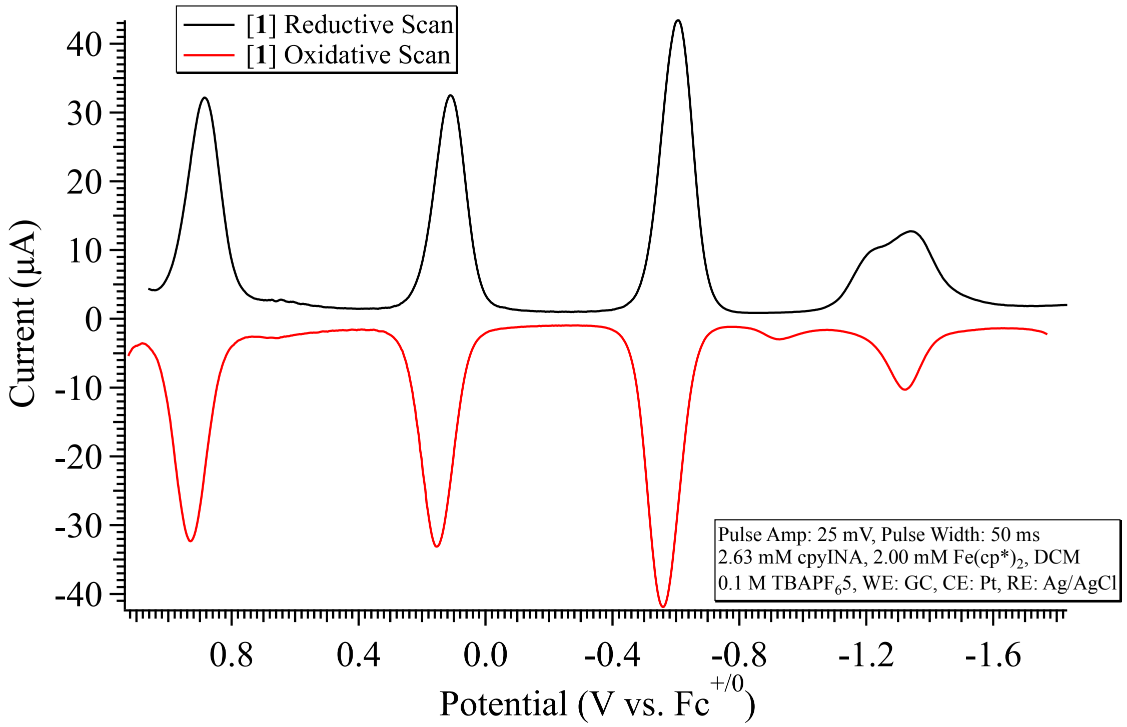

Fig. S20. Differential pulse voltammogram of **1** in DCM at analyte concentrations of 2.63 mM with 0.1 M $TBAPF_6$ and an internal standard of decamethylferrocene ($E_{1/2}$ = –0.58 V vs. $Fc^{+/0}$). DPV's were recorded using a pulse amplitude of 25 mV, a pulse width of 50 ms and referenced to the ferrocene +/0 redox couple.

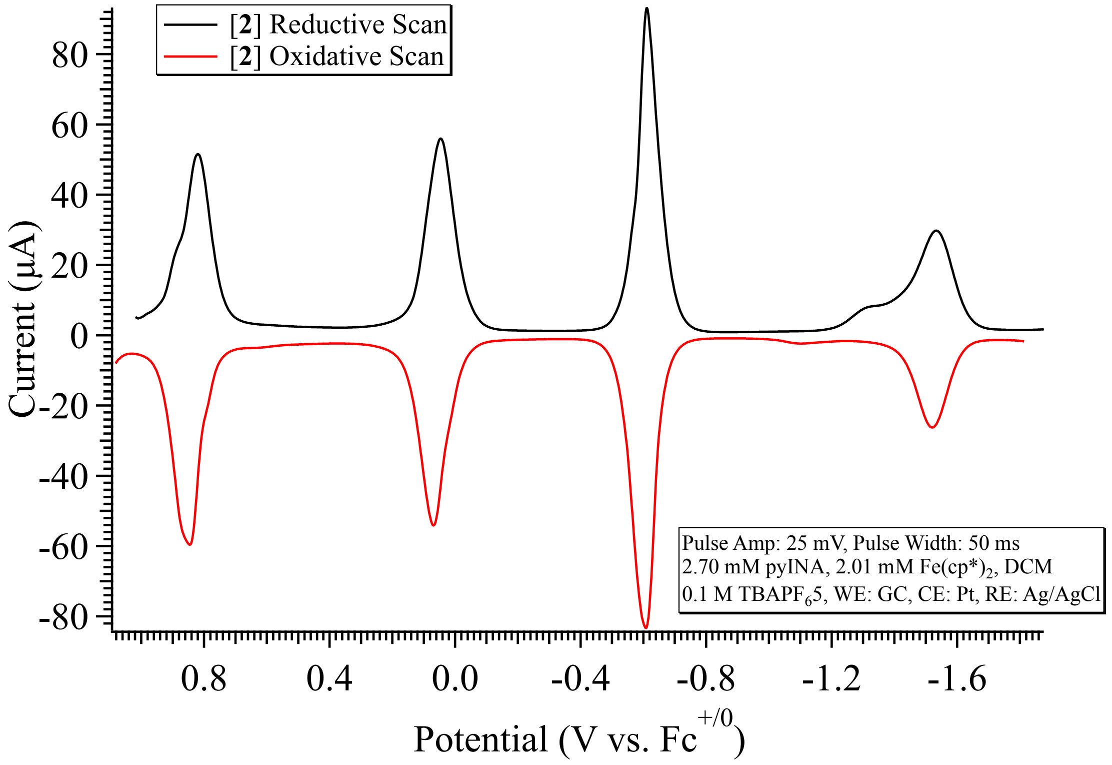

Fig. S21. Differential pulse voltammogram of **2** in DCM at analyte concentrations of 2.70 mM with 0.1 M $TBAPF_6$ and an internal standard of decamethyl ferrocene ($E_{1/2}$ = –0.58 V vs. $Fc^{+/0}$). DPV's were recorded using a pulse amplitude of 25 mV, a pulse width of 50 ms and referenced to the ferrocene +/0 redox couple.

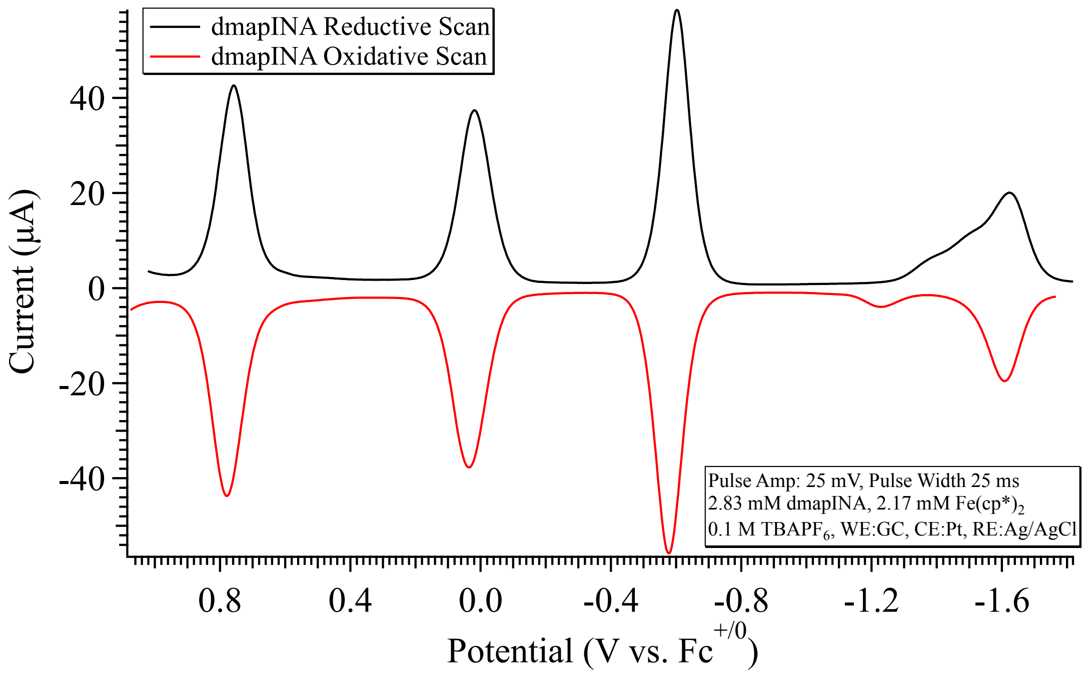

Fig. S22. Differential pulse voltammogram of **3** in DCM at analyte concentrations of 2.63 mM with 0.1 M $TBAPF_6$ and an internal standard of decamethyl ferrocene ($E_{1/2}$ = –0.58 V vs. $Fc^{+/0}$). DPV's were recorded using a pulse amplitude of 25 mV, a pulse width of 50 ms and referenced to the ferrocene +/0 redox couple.

Table S1. Integrated spectral areas and concentrations of ν(*CO*OH) bands for **1**–**3** in DCM obtained from fits presented in figure S9. Absorbance for 0.25 mM concentration was not large enough to determine spectral area for dimer band.

Complex	**Concentration** (mM)	A_m	A_d
**1**	1.825 (0.009)	2.59 (0.02)	0.61 (0.03)
	1.664 (0.006)	2.38 (0.02)	0.53 (0.02)
	1.417 (0.004)	2.10 (0.02)	0.42 (0.02)
	1.179 (0.003)	1.81 (0.02)	0.33 (0.02)
	0.960 (0.003)	1.50 (0.01)	0.24 (0.02)
	0.717 (0.002)	1.16 (0.01)	0.15 (0.01)
	0.483 (0.007)	0.814 (0.007)	0.086 (0.009)
	0.250 (0.003)	0.450 (0.006)	----
**2**	2.28 (0.01)	2.59 (0.02)	0.55 (0.03)
	2.130 (0.008)	2.47 (0.02)	0.49 (0.03)
	1.82 (0.01)	2.19 (0.02)	0.41 (0.02)
	1.534 (0.005)	1.89 (0.02)	0.31 (0.02)
	1.239 (0.004)	1.57 (0.01)	0.21 (0.02)
	0.942 (0.002)	1.24 (0.01)	0.14 (0.01)
	0.650 (0.003)	0.862 (0.008)	0.08 (0.01)
	0.317 (0.001)	0.440 (0.004)	---
**3**	2.20 (0.01)	2.51 (0.02)	0.55 (0.03)
	1.92 (0.02)	2.28 (0.02)	0.47 (0.02)
	1.676 (0.007)	2.06 (0.02)	0.38 (0.02)
	1.479 (0.005)	1.85 (0.02)	0.32 (0.02)
	1.230 (0.004)	1.61 (0.01)	0.23 (0.02)
	0.979 (0.004)	1.32 (0.01)	0.18 (0.01)
	0.762 (0.003)	1.042 (0.009)	0.13 (0.01)
	0.492 (0.002)	0.713 (0.006)	---

Table S2. Height of the monomeric band from absorption spectra of ($\mathbf{1}_2$)$^{2-}$ (612 nm); ($\mathbf{2}_2$)$^{2-}$ (487 nm); ($\mathbf{3}_2$)$^{2-}$ (550 nm) and concentrations for ($\mathbf{2}_2$)$^{2-}$ in THF with Co(cp*)$_2$ as a chemical reductant obtained from fits presented in figure S14.

**Complex**	**Concentration** (μM)	$\mathbf{H_m}$
($\mathbf{1}_2$)$^{2-}$	0.129 (0.004)	1.687
	0.094 (0.003)	1.372
	0.068 (0.003)	1.022
	0.049 (0.002)	0.761
	0.0030 (0.001)	0.498
($\mathbf{2}_2$)$^{2-}$	0.118 (0.002)	1.152
	0.090 (0.003)	0.940
	0.072 (0.003)	0.758
	0.041 (0.003)	0.476
	0.029 (0.002)	0.353
($\mathbf{3}_2$)$^{2-}$	0.132 (0.002)	1.026
	0.109 (0.003)	0.876
	0.081 (0.003)	0.679
	0.053 (0.003)	0.487
	0.029 (0.002)	0.294

Table S3. Measured dimerization constants of neutral (K_D) for **1–3** in DCM at 25 ºC.

$$\frac{[M]_0}{A_m} = \frac{1}{\varepsilon_m l} + \left(\frac{2K_d}{(\varepsilon_m l)^2}\right) A_m$$

$$\frac{2A_d}{[M]_0} = \varepsilon_d l - \left(\frac{\varepsilon_d l}{K_d}\right)^{1/2} \frac{(A_d)^{1/2}}{[M]_0}$$

**Complex**	**K** (monomer band, M^{-1})	**K** (dimer band, M^{-1})	**K** (average, M^{-1})
**1**	119 (6)	450 (70)	290 (40)
**2**	75 (5)	240 (90)	160 (50)
**3**	130 (8)	600 (200)	400 (100)

Table S4. Measured dimerization constants of neutral (K_D) for **1–3** in DCM at 25 ºC.

$$\frac{A_m}{[M]_0} = \varepsilon_m - \frac{K_d}{\varepsilon_m}\left(\frac{2(A_m)^2}{[M]_0}\right)$$

**Complex**	**K** (monomer band, M^{-1})
**1**	120 (7)
**2**	73 (5)
**3**	126 (9)

Table S5. Measured dimerization constants of dianionic (K_{2-}) for $(\mathbf{1}_2)^{2-}$–$(\mathbf{3}_2)^{2-}$ in THF at 25 ºC with $Co(cp^*)_2$ as a chemical reductant.

$$\frac{[M]_0}{H_m} = \frac{1}{\varepsilon_m l} + \left(\frac{2K_d}{(\varepsilon_m l)^2}\right) H_m$$

**Complex**	$\mathbf{K_{2-}}$ (monomer band, M^{-1})
$(\mathbf{1}_2)^{2-}$	2000 (400)
$(\mathbf{2}_2)^{2-}$	2200 (300)
$(\mathbf{3}_2)^{2-}$	2500 (300)

Table S6. Measured dimerization constants of dianionic (K_{2-}) for $(\mathbf{1}_2)^{2-}$–$(\mathbf{3}_2)^{2-}$ in THF at 25 ºC with $Co(cp^*)_2$ as a chemical reductant.

$$\frac{H_m}{[M]_0} = \varepsilon_m - \frac{K_d}{\varepsilon_m}\left(\frac{2(H_m)^2}{[M]_0}\right)$$

**Complex**	$\mathbf{K_{2-}}$ (monomer band, M^{-1})
$(\mathbf{1}_2)^{2-}$	2000 (400)
$(\mathbf{2}_2)^{2-}$	2200 (300)
$(\mathbf{3}_2)^{2-}$	2700 (300)
